# Differential effects of resource scarcity and pathogen prevalence on heterosexual women's facial masculinity preferences

**DOI:** 10.1017/ehs.2021.42

**Published:** 2021-09-16

**Authors:** S. Adil Saribay, Petr Tureček, Rüzgar Paluch, Karel Kleisner

**Affiliations:** 1 Department of Psychology, Kadir Has University, Istanbul, Turkey; 2 Department of Philosophy and History of Sciences, Faculty of Science, Charles University, Prague, Czech Republic; 3 Center for Theoretical Study, Charles University and Czech Academy of Sciences, Prague, Czech Republic; 4 Department of Clinical Psychology, Utrecht University, The Netherlands

**Keywords:** mate choice, facial preference, masculinity, femininity, resource scarcity, pathogen prevalence

## Abstract

The present research focused on how environmental harshness may affect heterosexual women's preferences of potential male mates’ facial characteristics, namely masculinity–femininity. The evidence on this issue is mixed and mostly from Western samples. We aimed to provide causal evidence using a sample of Turkish women and Turkish male faces. A video-based manipulation was developed to heighten environmental harshness perceptions. In the main experiment, participants were primed with resource scarcity, pathogen prevalence or neither (control). They then saw masculinised vs. feminised versions of the same faces and indicated the face that they would prefer for a long-term relationship and separately rated the faces on various dimensions. In general, masculinised faces were perceived as slightly more attractive, slightly healthier and much more formidable. A multilevel Bayesian model showed that pathogen prevalence lowered the preference for masculinised faces while resource scarcity weakly elevated it. The overall drop in attractiveness ratings in cases of high perceived pathogen prevalence, one of the strongest effects we observed, suggests that during epidemics, the formation of new relationships is not a favourable strategy. Implications for evolutionary theories of mate preference are discussed.

## Graphical abstract

**Figure:**
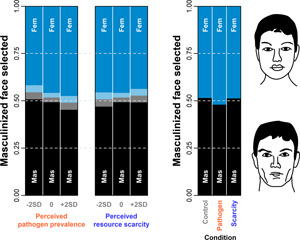

**Social media summary:** Environmental harshness may affect heterosexual women’s long-term partner preferences but the evidence on this issue is mixed and mostly from Western samples. We manipulated Turkish women’s perceptions of resource scarcity and pathogen prevalence independently and asked them to choose between masculinized and feminized versions of Turkish male faces for long-term partnership and to also to rate these faces on various qualities such as attractiveness. Pathogen prevalence lowered the preference for masculinized faces while resource scarcity weakly elevated it. Also, women who perceived higher pathogen prevalence found the male faces less attractive overall, suggesting that during epidemics, women may adopt a more conservative strategy in terms of forming new relationships.

Many factors are known to influence mate choice in human beings (see Pisanski & Feinberg, 2013; Puts, 2010, for an overview). The evidence for heterosexual women's preferences in male faces demonstrates a considerable amount of heterogeneity. Evidence exists for both greater preference for masculine (Cunningham et al., 1990; DeBruine et al., 2006; Grammer & Thornhill, 1994) and feminine (Little & Hancock, 2002; Perrett et al., 1998) features. This may stem from moderators or the different meanings of masculinity to different women and in different contexts. In fact, several moderators have been discovered, such as own mate value (Penton-Voak et al., 2003), relationship status (Little et al., 2002), current position in the menstrual cycle (Penton-Voak et al., 1999) and focus on short- or long-term mating (Little et al., 2002). Overall, the direction of these moderating effects suggests that male facial masculinity signals genetic quality to women. For instance, women prefer more masculine faces when they are fertile (Gangestad et al., 2004; Gildersleeve et al., 2013), when they are already partnered (Little et al., 2002) and when they are focused on short-term mating (Waynforth et al., 2005) – that is, in situations in which they would be expected to prioritise genetic quality over other aspects of a potential mate. It must be noted that some fertility measures appear to lack sufficient accuracy (Blake et al., 2016) and recent studies using superior (hormonal) measures have found no evidence for stronger masculinity preferences in women in the high fertility phase of the menstrual cycle (Dixson et al., 2018a, 2018b; B. C. Jones et al., 2018; Marcinkowska et al., 2018).

Likewise, women's masculinity ratings of male faces are positively correlated with attractiveness (Johnston et al., 2001; Penton-Voak & Perrett, 2000; Thornhill & Gangestad, 1999; Zuckerman et al., 1995). Yet this positive correlation is not consistent (DeBruine et al., 2006; Perrett et al., 1998; Swaddle & Reierson, 2002), perhaps because there are multiple costs and benefits communicated by facial masculinity (Marcinkowska et al., 2019). Consequently, it is not clear what a greater preference for male facial masculinity by heterosexual female perceivers really means.

The multiplicity in the cost and benefits of choosing masculine mates may indicate a trade-off between two female priorities: *quality* (genetic fitness and health) and parental *investment* (Gangestad & Simpson, 2000; Little et al., 2002). For instance, men with more masculine facial features are perceived as possessing higher quality genes (Thornhill & Gangestad, 1999) and health (Johnston et al., 2001) and in fact enjoy superior health and strength (Fink et al., 2007; Thornhill & Gangestad, 2006; but see Boothroyd et al., 2013). However, more masculine men are also perceived as less reliable investors in offspring and in fact have a greater number of short-term partners (Boothroyd et al., 2008; Perrett et al., 1998). Furthermore, in environments with heavy male-to-male competition, formidability (i.e. fighting ability), which overlaps with masculinity (e.g. Swaddle & Reierson, 2002), may signal that a male partner is able to acquire resources and protect kin, further complicating the issue.

## Mating context: short- vs. long-term relationships

The above-mentioned trade-off may depend on mating context. Women prefer more masculine male faces if they are oriented toward short- rather than long-term relationships (Waynforth et al., 2005; but see Stower et al., 2019). In the context of a short-term relationship, facial cues related to a male's paternal investment potential (vs. to his genetic fitness) may have less weight in driving preferences. In other words, women may value genetic quality more when they do not (vs. do) expect investment from a partner and their preferences for facial features may reflect this tendency. On the contrary, in the context of a long-term relationship, paternal investment and relationship commitment may become more important than genetic quality, increasing the attractiveness of male facial femininity as a signal of paternal investment potential (Little et al., 2002; Scheib, 2001; but see Luevano & Zebrowitz, 2007). In short, mating context is an important factor in the trade-off between quality and investment. However, this trade-off has to be resolved within a broader ecological context as we review next.

## Ecological factors: pathogen prevalence and resource scarcity

The majority of studies on mate selection have focused on two kinds of environmental harshness: resource scarcity (RS) and pathogen prevalence (PP). The former is expected to increase the risk of conflict and failure to meet physical needs (e.g. malnutrition) and the latter is expected to increase the risk of disease.

### Pathogen prevalence

Women have been found to show stronger preference for masculinity in cultures with higher PP. DeBruine et al. (2010) found that across 30 countries the lower the health of the nation, the higher the likelihood that women from that country preferred facial masculinity. This effect holds when accounting for other factors that correlate with national health and predict women's masculinity preferences, such as national income inequality and homicide rates (DeBruine et al., 2011).

To more directly test the effect of PP on facial preferences, a number of studies have used priming techniques (e.g. presenting images designed to heighten perceptions of PP). One such study found that priming women with thoughts of increased PP (vs. no priming) led them to show greater preference for masculine male faces (Little et al., 2011). Given the above-mentioned research suggesting that greater male facial masculinity is linked to higher genetic quality, this has been interpreted as a greater preference for quality over investment. That is, in pathogen-loaded environments, women appear to prioritise the quality of paternal genetic contribution over paternal investment in offspring and to implement this strategy via increased preference for male facial masculinity, which they presumably perceive (consciously or not) as positively related to quality and not related to (or negatively related to) investment potential. Thus, women may value masculinity primarily as a health (immunocompetence and current physical well-being) cue. In turn, as a signal of a mate's health, facial masculinity may become valued by women in pathogen-loaded environments because it increases the chances of survival of offspring bearing this mate's genes. Indeed, perceived and actual health are correlated positively with judgements of masculinity in male faces (Rhodes et al., 2003), and perceptions of health partially mediate the relationship between judgements of attractiveness and masculinity (Rhodes et al., 2007). Males with more masculine faces are also perceived by women as being less likely to invest in parenting (Kruger, 2006) and the relationship (O'Connor et al., 2012).

It must be noted that several studies have failed to find evidence that women's exposure to pathogen cues or self-reported pathogen (including COVID-19) sensitivity was related to their greater masculinity preferences in male faces, highlighting the need for further research on this topic (Clarkson et al., 2020; Dixson et al., 2017; McIntosh et al., 2017; Pazhoohi et al., 2021; Pereira et al., 2020).

### Resource scarcity

RS and PP tend to go together in natural environments. Thus, some of the earlier mentioned cross-cultural evidence on PP can also be taken as partially indicating effects of RS (Brooks et al., 2011; but see DeBruine et al., 2011). Experimental research has tested the independent effects of RS and PP. For instance, being primed with thoughts of resource scarcity (vs. abundance) leads women to prefer more feminine male faces in a long-term mating context (Little et al., 2007). This has been interpreted as a greater preference for investment over quality, given the research mentioned above suggesting that more masculine men tend to be perceived by women as less reliable investors in offspring (Kruger, 2006). That is, in resource-scarce environments, women appear to prioritise paternal investment in offspring over higher quality genes and implement this strategy via increased preference for male facial femininity. Likewise, another study found that exposing women to cues of societal resource abundance led to increased preference for male facial masculinity, while exposing them to cues of scarcity led to decreased preference for male facial masculinity, compared with both baseline preferences and a mixed-wealth (i.e. societal income inequality) condition (Little et al., 2013, Exp. 2). Thus, when resources are scarce, investment becomes a more pressing challenge and women appear to prefer mates with higher potential to invest in the offspring.

However, conflicting findings exist regarding the effects of RS. Lu et al. (2015) studied a large and varied sample of Chinese women and found that resource abundance was associated with women's greater preference for paternal investment (‘good father’) over quality (‘good genes’) and resource provision potential (‘good provider’) in males. They replicated this finding in an experiment. Dixson et al. (2017) found no relationship between level of urban development in three Melanesian islands and preference for masculine male faces of women inhabitants of those islands. Pettijohn et al. (2009) found a stronger self-reported preference for mature (vs. non-mature; akin to masculine vs. feminine, respectively) personality type in ideal mates in hungry (vs. satiated) women, taking hunger as a proxy for RS. These conflicting findings could be due to differences in samples or methods (self-report vs. face rating). In any case, they point to the need for further research.

Even if women were shown to have a greater preference for femininity under RS, this would be puzzling because RS should increase competition (Daly & Wilson, 2001) and male-to-male violence; and men perceived as more masculine, and hence more formidable, by women may actually have an advantage in male–male physical competition (Fink et al., 2007) as well as greater access to resources than feminine men. More masculine men may also be more likely to divide those resources among a greater number of mates and offspring and present a risk of intra-pair violence (Ryder et al., 2016; Snyder et al., 2011), partially offsetting the benefits of choosing them as mates.

Supporting this idea, faces signalling higher testosterone levels are preferred more strongly by women in resource-scarce countries (Moore et al., 2013); and increased awareness of male–male competition in the environment is related to increased preference for male facial masculinity (Brooks et al., 2011; Little et al., 2013; but see Marcinkowska et al., 2019). For instance, priming women with images depicting combative (vs. non-combative) male sports (Exp. 1a) or with images depicting weapons (vs. not; Exp. 1b) increased their preference for male facial masculinity (Little et al., 2013).

In sum, there seems to be a conflict between the idea that resource-scarce environments are laden with increased male–male violence and the idea that women prefer more feminine men in resource-scarce environments. For this reason, further research is necessary to more carefully examine mechanisms underlying greater preference for feminine mates under RS.

## The present research

Motivated by the observation of mixed findings and insufficient understanding of mechanisms, we aimed to provide experimental evidence regarding heterosexual women's preferences for male facial masculinity (vs. femininity) under environmental harshness. We focused on the long-term/committed mating context because past research (Little et al., 2007) has shown that this is where environmental harshness is likely to have an effect on mate preferences. We did not include a measure of position in ovulatory cycle as recent studies lead us to conclude that this is irrelevant (e.g. Marcinkowska et al., 2018).

Importantly, most studies reviewed above relied on samples from Western, educated, industrialised, rich, and democratic (WEIRD) cultures (Henrich et al., 2010). In a recent, well-designed study with a culturally wide sample, Marcinkowska et al. (2019) found that heterosexual women in *more* developed nations had a *stronger* preference for male facial masculinity (see also Scott et al., 2014). However, conflicting findings in the literature alert us to the possibility that results obtained from correlational and experimental studies could differ (e.g. A. L. Jones & Jaeger, 2019). Experimental studies with non-WEIRD samples are even less common and our goal was to conduct an experimental study on this topic in a non-WEIRD and predominantly Muslim culture.

*Perceived* environmental harshness should matter more than objective environmental indicators, which are hard for individuals to keep track of in modern, complex societies. Thus, in a pilot study (see Supporting Information, S1), we aimed to develop a manipulation that would temporarily heighten perceptions of RS and PP, separately, and to provide independent evidence of its effectiveness, a component that is lacking in most, if not all, of the studies reviewed above.

In the main experiment, primed with environmental harshness, participants were subsequently asked to choose between masculinised and feminised versions of male faces for long-term mating (our main dependent variable), and to rate the same faces on health, formidability and attractiveness. We use perceived health as an index of perceived genetic quality and attractiveness as a proxy for mate preferences. These ratings were included because, in our view, the literature has focused more on mate preferences but not enough on the reasons underlying such preferences. In one exception, Luevano and Zebrowitz (2007) used health, dominance and warmth ratings from previous databases and found that these did not explain women's greater preference (operationalised in terms of attractiveness ratings) for more masculine male faces in a short-term mating context in a new sample. Like those authors, we used facial ratings to shed light on the facial preferences of women in our study but collected those ratings from the same sample of participants, which is more suitable for testing mediation. We also collected self-report measures of environmental harshness perceptions for exploratory purposes.

Acknowledging the mixed evidence reviewed above, we anchored our hypotheses in the following directions: we expect that there is a non-random association between environmental conditions and masculinity preferences. Under environmental harshness, women should prefer masculine over feminine facial features in potential mates. In pathogen-prevalent environments, women should value masculinity primarily as a genetic quality cue. In resource-scarce environments, women should value masculinity primarily as a cue of formidability. Stated differently, health ratings should show a closer relation to mate choice and to attractiveness ratings in the PP condition, whereas formidability ratings should show a closer relation to mate choice and attractiveness ratings in the RS condition. This should help to clarify the ambiguity in the literature in terms of the reasons underlying women's facial preferences.

## Method

In a separate study, we developed an experimental manipulation intended to heighten (or not) perceptions of two types of environmental harshness (see Supporting Information, S1). Subsequently, in the main experiment (described below), we implemented this manipulation and then measured preferences for feminised vs. masculinised versions of the same faces in a two-alternative forced-choice task.

### Participants

We invited undergraduate heterosexual women enrolled in the subject pool at Boğaziçi University's Psychology Department. We recruited 385 participants for the experimental data collection; two were removed immediately for idiosyncratic reasons (one had participated in the pilot study and one was male), 65 participants were removed because they were non-heterosexual (12 bisexual, 47 homosexual, 6 other) and 24 were removed from the sample because they answered both questions of the video attention check questionnaire wrong. From the remaining sample of 298, we completely removed the data from nine raters who recognised more than four faces and another 110 data points where the face was recognised (21 people recognised one, 14 people two, 7 people three and 10 people four faces). The analyses are based on 3358 data points provided by the remaining 289 people (102 in control, 101 in PP, 86 in RS condition; mean age 21.03, SD 2.98). We present posterior distributions for the following alternative inclusion criteria in the Supporting Information S7: (a) include data points where the target was recognised; (b) include those who answered both questions wrong; and (c) include non-heterosexual respondents. Conclusions drawn from these alternatives are identical to conclusions discussed in the main article.

### Materials and procedure

#### Manipulation

Participants were randomly assigned to one of three conditions – PP, RS and control. After being given a brief overview of the study and providing content, they were immediately shown the video corresponding to the condition they were assigned to. The videos were narrated and contained dramatic images and ostensible facts relevant to either PP or RS. The control video contained information about space and planets. See see Supporting Information, S1 for further details.

#### Facial preference task

We measured women's preferences for facial masculinity (vs. femininity) using a two-alternative forced-choice task with 12 trials. In each of 12 trials, a relatively masculine and a relatively feminine version of the same face were presented together on the computer screen. Participants were asked to choose the face that they would prefer for a long-term relationship.

To prepare these stimuli, we first selected a set of 20 male and 20 female facial photographs from the Bogazici Face Database (Saribay et al., 2018). The face bearers and the participants of this study are from the same age group (university students). All photographs were standardised and the targets displayed a neutral facial expression. We selected the male facial photographs quasi-randomly from a set of targets who were more likely to have graduated from the university, in order to decrease the chance of recognition; the female photographs were selected completely randomly from the entire database. Using webmorph (DeBruine & Tiddeman, 2017), the 20 photographs were averaged separately to obtain one male and one female average. We then selected a set of 12 male faces randomly within the set of 20 used for obtaining this average for use in the experiment. We placed these averages on the opposite ends of a single dimension and transformed each of the 12 faces toward both ends by 50%. That is, for each of the 12 faces, we obtained one version that was 50% more masculine (more like the average of 20 males) and another version that was 50% more feminine (more like the average of 20 females).

In the facial preference trials presented to participants, we counterbalanced the location on the screen (left–right) of masculine vs. feminine versions the 12 faces. That is, for about half the participants (randomly assigned), masculine faces appeared on the left side of the screen throughout this task and for the remaining participants masculine faces appeared on the right side of the screen.

#### Video attention check questionnaire

Participants were asked the same video-specific factual questions as in the pilot study (see Supporting Information, S1) to check that they had attended to the content of the video. As stated above, 24 participants who answered both questions wrongly were removed.

#### Facial ratings

In three blocks, whose order of presentation was randomised, participants were shown the 24 faces (both the masculinised and feminised version of the 12 faces) that they encountered in the facial preference task. Each face was presented individually and the order of faces was individually randomised. The blocks corresponded to the rating dimension – attractiveness, health and formidability. Participants were asked to rate how attractive/healthy/formidable they found each face using a 1 (very low) to 7 (very high) rating scale.

#### Video impact scale

To assess the overall effect of watching the video, three items (e.g. ‘Watching the video made me feel worried’) were administered (see Supporting Information, S1). Figure S1 in the Supporting Information demonstrates that participants in both experimental conditions gave higher ratings than those in the control condition.

#### Environmental harshness perceptions

For exploratory purposes, we obtained self-reported perceptions of PP and RS, also used in the pilot study (see Supporting Information, S2 for the full list of items). These were administered in randomised blocks. Both scales had sufficient internal consistency, Cronbach's *α* = 0.84 for PP and 0.71 for RS. They also provide some (although imperfect) evidence of the intended effect of the manipulation (see Supporting Information, Figure S2 and the discussion therein). See Supporting Information, S3 for a factor analysis of scale items and Supporting Information, S1 for some analyses with subscales.

#### Demographics

Participants were asked to report their age, sexual orientation, relationship status (109 in an ongoing relationship, 180 without a partner) and their own perceived mate value separately for their face (mean = 4.58, SD = 1.16) and body (mean = 4.55, SD = 1.32). The correlation between face and body mate value was *r* = 0.64.

#### Face recognition

Participants were shown the original (non-transformed) version of the 12 faces and asked to report whether they recognise the face-bearer (i.e. they have seen the person in real life) or not. As stated above, these responses were used to remove data points which could be contaminated by personal knowledge about the face-bearers.

#### Debriefing

Participants were asked four questions that probed their thoughts about the experiment, including whether they followed a particular strategy in the facial preference task and how they thought the priming video may have affected them.

### Statistical analysis

**Figure 1.**
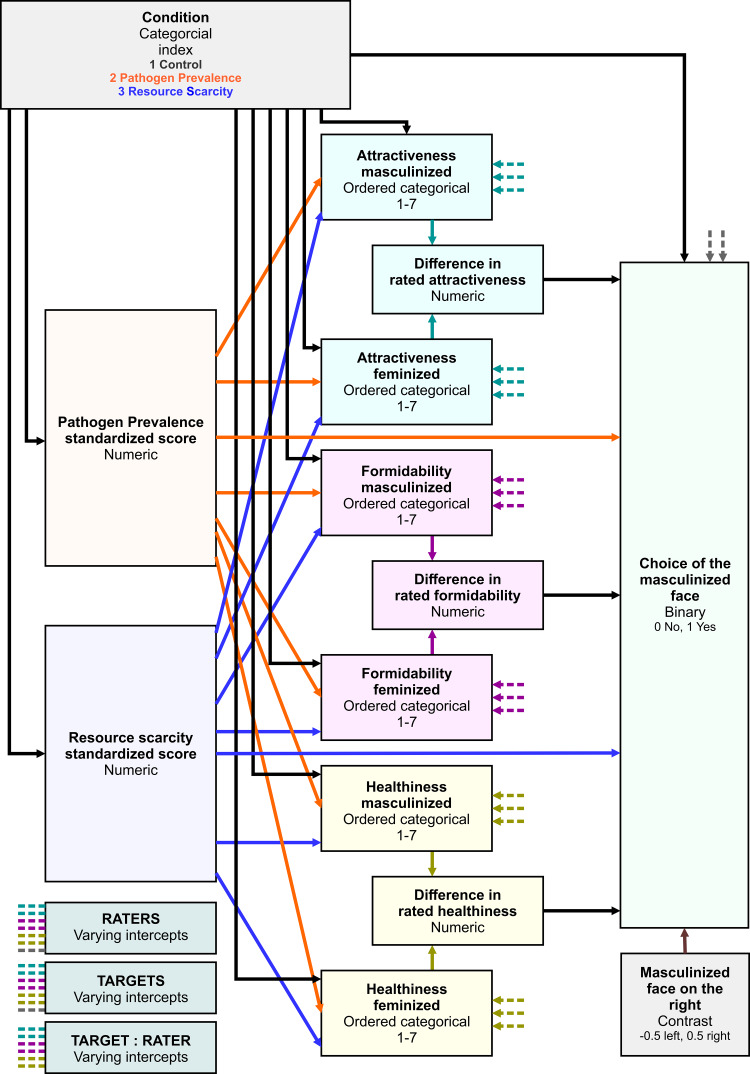
Model structure. Black arrows from the Condition variable represent sets of three parameters, one for each condition.

We employed a Bayesian inference to evaluate posterior distribution of parameter values in a mediation model with multilevel data structure in Figure 1. We used the choice of the masculinised version of the target photograph as an ultimate binary response variable. The probability that the participant indicates the masculinised face as a more suitable long-term partner (*p*) and the complementary probability of the opposite choice (*p* − 1) were linked to linear predictors using a log-odds link function
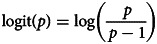
For each condition (control, PP, RS) an independent intercept of a baseline choice probability was calculated. Differences in rated attractiveness, formidability and healthiness entered the logistic regression with an independent slope in each condition. Perceived PP and RS scores (both standardised before the analysis) entered the regression as continuous linear predictors. The last predictor in the logistic regression was a contrast of the location of the masculinised photo administration (if the masculinised photo was placed on the left side of the monitor, this variable assumed to have a value of −0.5; if it was placed on the right side of the monitor, it assumed to have a value of 0.5). Varying intercepts, one for each target, were drawn from a normal distribution characterised by a hyperparameter *σ*_t_ corresponding to its standard deviation, and varying intercepts for raters were drawn from a normal distribution characterised similarly by *σ*_r_.

**Figure 2.**
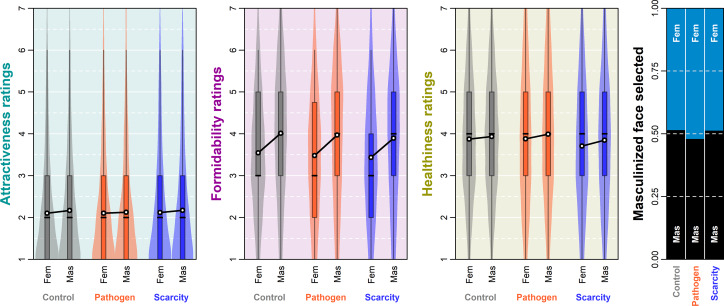
Raw data distributions. Boxes span the interquartile intervals, whiskers indicate intervals containing 95% of all ratings, the thick horizontal line within each box shows the median and the white dot shows the arithmetic mean of the distribution.

To investigate a potential mediation effect of condition through changes in perceived attractiveness, formidability or healthiness, ordinal logistic regressions with a cumulative-logit link function were used. Each rating of the masculinised and the feminised version of the face (see the structural model in Figure 2) was predicted from the condition, perceived pathogen prevalence, perceived resource scarcity and varying intercept of the target face, rater and interaction between target and rater. For each characteristic rated on a Likert scale (1–7), a common set of six cut-points was evaluated slicing the continuum with ordered thresholds corresponding to log-odds probability of scoring above given threshold. Baseline log-odds probabilities given by common cut-points were modified with linear predictors *ϕ*_mas_ for masculinised and *ϕ*_fem_ for feminised photo. Slopes corresponding to conditions, standardised PP and RS scores were estimated for *ϕ*_mas_ and *ϕ*_fem_ independently. Varying intercepts, one for each target, rater and interaction between target and rater (since certain raters might find certain faces especially appealing/unappealing regardless of masculinisation/feminisation) common for *ϕ*_mas_ and *ϕ*_fem_ were drawn from three independent normal distributions with zero mean and respective standard deviations. Posterior distribution of differences between ratings was calculated from the posterior distributions of masculinised and feminised ratings within rater–target pairs.

For the analysis of mediation to be complete, rater's standardised perceived PP and RS scores entered the analysis not only as predictors but also as dependent variables predicted by priming condition. An independent intercept was estimated for each combination of condition and scale in the same way that these intercepts were estimated in the pilot study.

The whole model expressed as a set of equations can be found in Supporting Information, S5. Unbiased, weakly regularised priors were used for all parameters and hyperparameters (see Supporting Information, S5 for numeric and S6 for graphical representations of prior distributions). Joint posterior distribution of all model parameters was sampled with the ulam() function from the rethinking R package (McElreath, 2020) using Stan's (Stan Development Team, 2018) MCMC (Markov Chain Mote Carlo) infrastructure. Ten thousand samples were extracted from the posterior distribution using the extract.samples() function. Distributions of differences between ratings and contrasts between conditions that were not represented by model parameters directly were calculated from these samples. To illustrate the model's predictions and translate changes in log-odds into probabilities, contrafactual plots and estimates were obtained linking a simulated data with the sampled posterior parameter distribution using the link() function. Results were visualised with custom-built functions using base R graphics. The analysis was conducted using the R version 3.6.2. The data and the analytic code that support the findings of this are openly available at https://github.com/costlysignalling/EnviroHarsh. The model structure is displayed in Figure 1.

## Results

### Association of pathogen prevalence and resource scarcity with the probability of masculinised face selection

Overall, a masculinised face was indicated as a more suitable long-term partner in 50.2% of all choices (see the raw choice overview in Figure 2 and Table S3). The preference for masculinised face was lowest under PP priming. The posterior distribution of log-odds difference between control and PP condition was 0.17 (89% CI 0.02, 0.33), it was 0.04 (89% CI −0.12, 0.20) between control and RS and 0.13 (89% CI −0.03, 0.30) between RS and PP. Masculinised face presented on the right side of the monitor was selected with probability 0.51 (89% CI 0.50, 0.53) while masculinised face presented on the left side was selected with probability 0.49 (89% CI 0.47, 0.50), all else being equal. The comparison of both effects using counterfactual plots can be found in Supporting Information, S8, Figure S8.

For each standard deviation on the perceived PP scale, the log-odds of masculinised face selection as a long-term partner decreased by 0.06 (89% CI 0.00, 0.12). Perceived RS had little impact on the preference for facial masculinity. The impact of both scales on masculinised face selection probability is illustrated in Supporting Information, S8, Figure S9.

### Association of perceived facial characteristics with probability of selecting the face

The difference in perceived attractiveness influenced the partner choice in all three conditions. The face that got a rating higher by 1 unit on the rating scale received on average a 0.10 bonus (89% CI −0.02, 0.23) to the log-odds of being selected as a more suitable partner in the control condition (0.09 in PP and 0.06 in RS condition). On the other hand, as Figure 2 shows, our participants rated the faces quite low in attractiveness on average, and this may partly be responsible for the small effect sizes that we observed in terms of mating preferences.

The difference in perceived formidability had little impact on the choice (89% CI of the slopes in all three conditions were within the range −0.12, 0.12). Similarly, the difference in perceived healthiness seemed to play little if any role in control and PP conditions (see Supporting Information, S8, Figure S10). In the RS condition, this slope was, however, most likely positive with mean 0.07 (89% CI −0.02, 0.16). The standard deviation of varying intercepts of targets (mean 0.72, 89% CI 0.50, 1.03) was much higher than the standard deviation of varying intercepts of raters (mean 0.12, 89% CI 0.01, 0.25), i.e. the selection was driven by how masculinisation or feminisation suited respective faces. The differences in systematic preferences for either manipulation between raters were much smaller. For discussion of the model's predictive accuracy and the role of varying intercepts in it, see Supporting Information, S10.

Distributions of raw ratings are illustrated in Figure 2 (numerical summaries of distribution means including standard deviations can be found in Supporting Information, Table S3). The masculinised version of the target photograph was likely to be rated as more attractive in the control (expected difference between ratings 0.06 [89% CI 0.03, 0.10]) and RS conditions (0.04 [89% CI 0.00, 0.08]). In PP condition, this difference was, however, far from conclusively positive (0.02 [89% CI −0.02, 0.06]). Varying intercepts of targets (SD 5.97, 89% CI 4.26, 8.15) explained most of the rating variation. Varying intercepts of raters showed lower variation (SD 3.00, 89% CI 2.75, 3.25) and so did varying intercepts of target–rater interactions (SD 2.48, 89% CI 2.35, 2.61), which altogether points to good agreement between raters when it comes to attractiveness. The differences between expected mean ratings across conditions were negligible. Participants with high self-report PP scores showed a tendency to use overall lower attractiveness ratings (see Supporting Information, S11).

Masculinised faces were rated as substantially more formidable than their feminised versions. The expected mean rating difference was 0.44 (89% CI 0.39, 0.49) in control, 0.47 (89% CI 0.42, 0.52) in the PP condition and 0.42 (89% CI 0.37, 0.48) in the RS condition. Portions of rating variability attributable to the differences between targets and between raters were quite balanced. Variation between raters mattered the most (SD 2.07, 89% CI 1.91, 2.24) followed closely by the varying intercepts of target–rater interaction (SD 1.90, 89% CI 1.81, 1.99) and target varying intercepts coming last (SD 1.43, 89% CI 1.00, 2.04). Formidability, unlike beauty, was in the eye of the beholder, relatively speaking.

**Figure 3.**
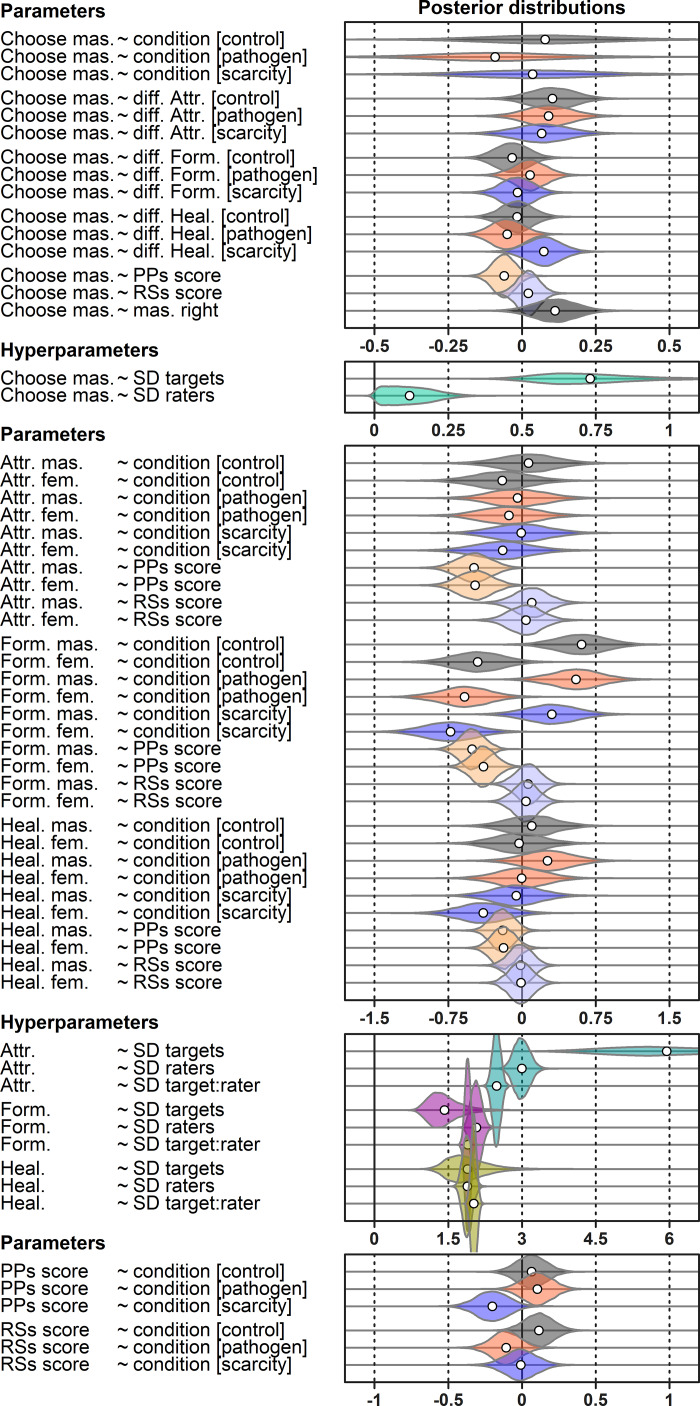
Posterior distribution of model's parameter values. Parameters correspond to arrows in Figure 1. The dependent variable is on the left and the predictor on the right side of the ‘~’. The white dot indicates the mean of sampled parameter values in the posterior.

Masculinised faces were also rated as healthier in all three conditions. The expected mean rating difference was 0.05 (89% CI 0.00, 0.10) in the control condition, 0.11 (89% CI 0.06, 0.16) in the PP condition and 0.14 (89% CI 0.08, 0.19) in the RS condition. The contributions of targets’ (SD 1.88, 89% CI 1.34, 2.62), raters’ (SD 1.89, 89% CI 1.73, 2.04) and target–rater interactions’ (SD 2.02, 89% CI 1.92, 2.11) varying intercepts to rated healthiness were levelled. Predicted distributions of ratings and differences between them are summarised in Supporting Information, S9, Figure S11. A summary of joint posterior distribution of model's parameter values can be found in Figure 3.

In sum, our findings offer evidence that a higher level of facial masculinity is preferred when the environmental harshness stems from resource scarcity while a lower level of facial masculinity is preferred in environments with prevalent pathogens. Indeed, this pattern emerges in several independent parts of our structural model: (1) in the condition with PP priming, masculinised faces are selected as potential long-term partners less frequently than in the other two conditions; (2) higher perceived PP scores are associated with lower preference for masculinised faces; and (3) in the PP condition, the expected difference in rated attractiveness between masculinised and feminised photograph is the lowest. The slight preference for facial masculinity seems to be a baseline that has a higher capacity to be influenced by PP than RS. Overall, the effects of masculinisation are rather small; the differences in attractiveness as well as the impact of masculinisation/feminisation on target attractiveness are majorly driven by differences between targets. The mediation paths from the condition through perceived PP and changes in rated attractiveness are present, although not very important. If we run a simplified model without mediation to evaluate the total effect of priming, we get a result that is not very different from priming's direct effect in the structural model (see also Figures S13 and S14 in Supporting Information S10).

## Discussion

The present research sought to test the effects of two types of temporarily heightened environmental harshness perceptions – pathogen prevalence and resource scarcity – on heterosexual women's preferences for relatively masculine vs. feminine male faces, a subject on which there is mixed evidence in the literature. We developed a strong, video-based manipulation method and conducted a pilot study to test its effectiveness, which is a component not present in most, if not all, of the earlier work. While the evidence was not unequivocally supportive, we interpreted it as indicating the feasibility of our manipulation. In the main experiment, we primed environmental harshness perceptions using these videos and used masculinised vs. feminised versions of the same faces to test heterosexual women's preferences in a long-term relationship context. We also collected continuous ratings of the faces and self-reported pathogen prevalence and resource scarcity perceptions as additional information. Our experimental study went outside the typical Western populations and collected data from Turkish women in the hopes of contributing to the recent efforts to diversify the database of psychological science.

### Evaluation of hypotheses

We predicted first that under environmental harshness, women would prefer masculine over feminine facial features in potential mates. Our findings indicate that this depended on the type of harshness, providing mixed support for this prediction: experimentally heightened perception of resource scarcity elevated the preference for facial masculinisation, although by a very small amount. However, experimentally heightened perception of pathogen prevalence *lowered* the preference for masculinised faces. These findings should be interpreted in light of the potential limitations of the experimental priming procedure, which we attempted to asses partially using perceived self-reported harshness following the priming. Priming conditions had little but not entirely negligible effects on perceived pathogen prevalence and resource scarcity scores. The manipulation worked as expected for pathogen prevalence (highest self-reported score in the pathogen prevalence condition vs. the other two conditions), whereas, unexpectedly, perceived resource scarcity scores were highest in the control condition. There may be many reasons why this happened. For instance, videos about pandemics and financial crises alike have the capacity to put participants’ resource scarcity into perspective, eliciting relative resource abundance, resulting in the surprising pattern we observed.

Next, we predicted that under pathogen prevalence, women would value masculinity in potential mates primarily as a genetic quality cue whereas under resource scarcity, they would value masculinity primarily as a cue of formidability. We tested this by examining whether healthiness ratings showed a closer relation to mate choice and to attractiveness ratings in the PP condition; and whether formidability ratings showed a closer relation to mate choice and attractiveness ratings in the RS condition. In general, masculinised faces were perceived as slightly more attractive, slightly healthier and much more formidable. Both rated formidability and healthiness were good predictors of rated attractiveness. Healthiness was the more important predictor of the two. There were virtually no differences between conditions in these relationships, however. Likewise, difference in perceived formidability and healthiness had little, if any, impact on mate choice overall. Thus, the safest conclusion for the time being is that there was no support for this prediction.

### Additional findings

Some additional findings may shed light on the conclusions reached above or be of general interest. First, the sources of perceptions of attractiveness, healthiness and formidability may differ. Beauty is in the face of the target rather than the eye of the beholder: In attractiveness ratings, differences between targets were much larger than differences between raters or target by rater combinations. The attribution of formidability was driven rather by differences between raters. In healthiness, the variation explained by raters and targets is balanced.

As stated before, the priming does not really change how the healthiness and formidability contribute to the rated attractiveness. Instead, perceived healthiness and formidability change together with rated attractiveness (see also Supporting Information, S12). The attractiveness ratings, however, seem to be influenced by the priming and perceived pathogen prevalence and resource scarcity the most. The overall drop in attractiveness ratings in cases of high perceived pathogen prevalence, one of the strongest effects we observed, suggests that during epidemics, the formation of new relationships is not a favourable strategy. New social contacts elevate the immediate probability of infection. Starting a new relationship under such circumstances is not worth the risk, especially considering the chance that the intention to form a long-term bond does not turn out to be mutual. More masculine men consistently show a stronger inclination to form uncommitted relationships (Polo et al., 2019; but see Bártová et al., 2020), so perhaps this trend contributes to the lower preference for masculinised face in PP condition and in individuals with high perceived PP regardless of the condition. The independent contribution of perceived PP score and PP priming indicates that the priming effect is not exhausted by changes in reported perceived environmental harshness. Still, all of these effects are not very strong. Most variation in rated attractiveness is between targets, which is not true in the case of formidability or healthiness. People seem to be well adapted to discriminate between consensually attractive and unattractive potential long-term partners. Healthiness and formidability, although indisputably important features of a desirable mate, are apparently not the only predictors of facial attractiveness. Environmental harshness, especially pathogen prevalence, has, however, the capacity to modify individuals’ mating behaviour. Individuals susceptible to infection (those with higher perceived PP) and all individuals during temporarily elevated PP are expected to show lower overall pursuit of new relationships (everyone seems less attractive to them) and prefer partners with facial features corresponding to higher expected level of relationship commitment (the balance shifts in favour of feminised faces). This psychological mechanism might be a result of an adaptation, because during epidemics, the balance of costs and benefits shifts in favour of a more restricted mating strategy (Alexopoulos et al., 2021).

Next, high perceived pathogen prevalence was associated with lower ratings in general, especially ratings of attractiveness and formidability, whereas perceived resource scarcity score was not related to the facial ratings. Resource scarcity was probably the status quo in our evolutionary history (Harari, 2014). Differently, high pathogen prevalence seems to be the temporary game-changer. In this latter sense, our findings are in accordance with the immunocompetence handicap hypothesis providing that facial masculinity is a costly signal. Facial masculinity is preferred when the benefits of masculinity (such as formidability, good genes, etc.) outweigh the costs of the immunosuppressive effects of testosterone. True, masculine facial features testify about high immunocompetence during childhood, but high testosterone levels (and its immunosuppressive effect) do not change overnight. Extrapolating on the immunocompetence handicap hypothesis (e.g. Rantala et al., 2012), when pathogen prevalence increases, high-testosterone males might be the most compromised and this might be more apparent with increasing age.

### Limitations and future directions

There are other limitations of our work apart from those noted above. Individual differences in women's sociosexuality may be related to facial masculinity preferences. For instance, Marcinkowska et al. (2019) found that heterosexual women who were less restricted sexually were more likely to prefer masculine faces in a forced-choice task similar to ours. This relation is not tested sufficiently in the literature and our study also did not include a measure of sociosexuality.

Neither our conceptual review nor our empirical methods distinguish sufficiently between genetic quality (heritable health) and current health. Distinguishing these conceptually and clarifying the relation between each and facial masculinity is a worthy direction for future research (Luevano & Zebrowitz, 2007), as it would help to further clarify the psychological meaning of male facial masculinity for women and its potential adaptive role, if any.

Finally, we have made an effort to improve on existing manipulation and measurement methods. This has made us more aware that further work is needed on these fronts if the literature is to arrive at replicable or more convergent findings on the topic at hand. Part of the reason for mixed evidence may be reliance on non-validated manipulations or psychometrically inferior measures of the central constructs, especially perceptions of pathogen prevalence and resource scarcity.

## Conclusion

It should be pointed out that the present research was conducted prior to the start of the COVID-19 pandemic. If, as the present experiment suggests, even a single priming video eliciting higher pathogen prevalence influences preferences in artificial situations like the forced choice between two rather similar versions of a single target, the actual impact of an ongoing pandemic on a real mate choice might be considerable.

## Supplementary material

For supplementary material accompanying this paper visit https://doi.org/10.1017/ehs.2021.42. click here to view supplementary material

## Research transparency and reproducibility

The data and the analytic code that support the findings of this are openly available at https://github.com/costlysignalling/EnviroHarsh
