# Supplementary material for: Differential effects of resource scarcity and pathogen prevalence on heterosexual women's facial masculinity preferences
Source: Evol Hum Sci. 2021 Sep 16;3:e48. doi: 10.1017/ehs.2021.42 (PMC10427302; doi:10.1017/ehs.2021.42)
Supplement: Supplementary file 1 [file S2513843X21000426sup001.docx]

Supplementary materials

**S1: Pilot Study (Development of the Experimental Manipulation)**

This section describes an earlier, separate study we conducted for the purpose of testing the experimental manipulation used in the main experiment. In addition, it contains some findings from the main experiment that rely on the same materials as the pilot study to test the effectiveness of the manipulation.

**Participants**

We attempted to collect as many participants as possible throughout a single academic semester. We included men in the sample for three reasons: We did not expect different effects of the videos for men versus women; we wanted to develop a manipulation that could be used with mixed-gender samples in the future; and we wanted to maximize the sample size.

In total 115 undergraduate participants were recruited for the pilot study in return for course credit. Six participants who failed to correctly answer both questions of the video attention check questionnaire (see Materials and Procedure) were removed from the sample, 22 non-heterosexual participants (5 bisexual, 10 homosexual, 7 other) were also removed for better comparison with the actual study, where opposite-sex mate choice is investigated. The remaining sample of 87 people consisted of 25 participants in Control, 31 in PP, and 31 in RS condition (49 women, *M*_age_ = 20.53, *SD*_age_ = 3.20).

**Materials and Procedure**

After examining manipulations of environmental harshness perceptions used in earlier studies (see references in the main article), we decided that a narrated video had the best chance of manipulating such perceptions. We developed three videos corresponding to the conditions of the planned experiment—pathogen prevalence (PP), resource scarcity (RS), and control. For the control conditions, we chose space as a neutral topic.

Each video was composed of clips available on a public video sharing website. The source of these clips varied (National Geographic, BBC, etc.). Each video was narrated by the same woman. We strived to create a professional presentation in which the images matched, as much as possible, the content of the narration. The narration content was created by the current research team and contained facts, some of which were exaggerated for effect, relevant to the topic of the experimental condition. Both local and global facts were provided. For instance, the PP video stated that “according to data from 2016 provided by the Ministry of Health, contagious diseases such as avian and swine flu affected 28% of the population of Turkey.” Similarly, the RS video stated that “In the past 50 years in Turkey, 36 of the important freshwater sources including [3 familiar and relatively large Turkish lakes] dried up completely.” The latter video focused on natural resources, water, and food, rather than economic resources per se. The video for the control condition was about space and talked about planets, dwarf planets, and natural satellites in the solar system.

Upon arrival in the laboratory, participants were escorted to isolated computer cubicles. They were randomly assigned to one of three conditions (PP, RS, control) and warned that they were about to be shown a video that may affect their mood negatively. No participant objected to the procedure and all were shown the video corresponding to the condition they were assigned to. Duration of the videos was about 90 seconds.

Once the video was finished, participants were asked a series of questions designed to assess the effect of viewing the video. To measure the general impact of the video, we designed a 3-item *video impact scale* whose content reflected the intended common effect of the PP and RS videos: “I found the video very impressive;” “I think what the video conveyed was very important;” and “Watching the video made me feel worried.” Responses were collected on a 7-point Likert-type scale (1 = “strongly disagree” to 7 = “strongly agree”). We expected the mean score from these three items to be greater for the experimental conditions compared to the control condition.

Beyond this expected general impact, videos were expected to have domain-specific effects. We attempted to capture these effects with two scales specifically designed to measure PP and RS perceptions (see Supplement S2 for the full list of items). The *pathogen prevalence scale* was based on the Perceived Vulnerability to Disease Scale (Duncan et al., 2009). The scale was translated to Turkish and back-translated. We modified some of the existing items (e.g., an outdated item involving a public phone was modified to be about public transportation) and added three new ones to make the scale more applicable to undergraduate students. The final version had 18 items. Duncan et al. (2009) reported two factors for this scale: perceived infectability and germ aversion and our data were consistent with this.

The *resource scarcity scale* was developed by the present authors for this study and had 16 items. In both scales, participants responded using a 7-point (1 = “strongly disagree” to 7 = “strongly agree”) Likert-type scale. An EFA with maximum likelihood extraction and promax rotation revealed three factors. Examining the content of items suggested that the first factor emphasizes worry about the future; the second one the felt need to act against an impending ecological crisis; and the third perception of the limited nature of resources.

We administered both scales in all conditions of the experiment (in random order; i.e., PP scale appeared before RS scale for about half of participants and this order was reversed for the remaining participants). Appropriate items were reverse-coded and a mean score was computed for each scale. Both scales had sufficient reliability, Cronbach’s α = .85 for PP and .80 for RS. We expected to see higher scores in the relevant condition compared to the other two conditions (e.g., PP scale scores should be higher in the PP condition than the other two conditions).

Following these two scales, participants were asked two questions that attempted to assess how much attention they paid to the video. These *video attention check questions* were asked because in the absence of attention to the video, we would not expect the video to have any impact on thoughts and perceptions (as assessed by the scales above). As the content of the videos were unique, the questions needed to be condition-specific. A sample item (from the PP condition) is as follows: “In the video you just viewed, which species was the crowd of animals before the image of the airplane?” The choices provided were “chicken” and “sheep.” We coded each response as correct or incorrect.

We were concerned that the type of item and response format employed in the measures above would fail to reflect the effect of the manipulation, as they often are found to (Talhelm et al., 2015; Yilmaz & Saribay, 2017). Thus, as a final way to assess the effectiveness of the manipulation, we requested a brief answer to a single open-ended question (“How did the video affect you?”).

Participants were shown two videos to compensate for the possible negative effects of the videos—the first one presenting positive facts (e.g., increased accessibility of knowledge across the globe via the internet) and the second one presenting clips with positive emotional tone (e.g., cute puppies playing). Finally, they were asked to complete a brief demographic form (e.g., age, gender, sexual orientation), debriefed, and thanked.

**Results and Discussion**

We employed a Bayesian inference to evaluate posterior distribution of parameter values in a simple model with multiple intercepts, one for each condition. Unbiased vague priors were used (normal distribution with mean 0 and standard deviation 1 for each intercept and exponential distribution with rate 1 for each standard deviation). Differences between conditions were based on 10,000 samples from the joint posterior distribution. The posterior distribution was explored using the ulam() function from the rethinking R package (McElreath, 2020) , samples were extracted using the extract.samples() from the same package. Condition influence on video impact and perceived RS and PP (scale scores) were investigated. Posterior distributions are reported for the pilot data, data from the actual experiment (perceived PP and RS estimates are extracted from the full structural model below), and both datasets analysed together.

First, we compared the mean of three video impact scale items across the three conditions. We expected participants in both experimental conditions to be more negatively impacted by the video exposure and thus, to have higher scores on this measure than the control condition participants. As seen in Figure 1, the mean scores in both experimental conditions tended to be higher than the same score in the control condition: the expected difference between PP and Control was 0.67 [89% CI: 0.23, 1.12] and the expected difference between RS and Control was 1.15 [89% CI: 0.70, 1.59]. When informed with the additional data collected during the subsequent experiment, the posterior distributions of differences between manipulation and control tighten around mean 0.91 [89% CI: 0.70, 1.13] for PP and 1.43 [89% CI: 1.21, 1.65] for RS condition (see Figure 1).


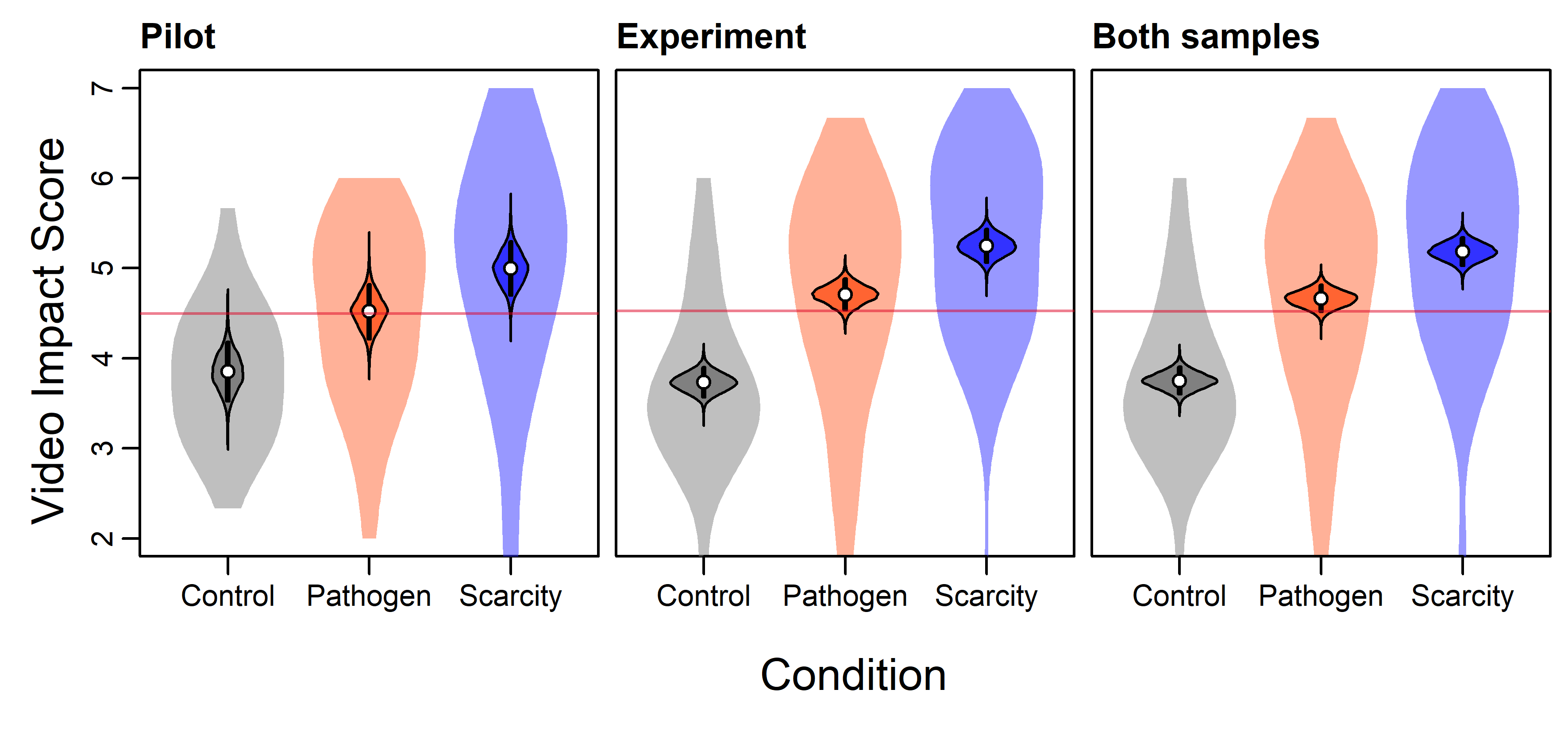


*Figure 1. Video impact scale scores (calculated on a scale of standardized sum of three items, shown on a scale of average item) across conditions and studies. Semi-transparent violin plots outline the raw data distributions, contoured violin plots show the intercept posterior distributions, black rectangles span 89% of these distributions, and white points indicate their means. The horizontal pink line indicates the grand mean.*

Second, we sought to test the specific effect of the videos using the environmental harshness perception scales.  Even though responses on such scales often fail to reflect the effect of situational primes (Talhelm et al., 2015; Yilmaz & Saribay, 2017), we explored whether they would support the specific effects of the videos.  We expected that mean PP scores would be higher in the PP condition compared to the other two conditions; and the mean RS scores would be higher in the RS condition compared to the other two conditions.

As seen in Figure 2, mean PP scale scores tended to be higher in the PP condition than both of the other conditions: the mean difference between PP and Control conditions is 0.59 [89% CI: 0.22, 0.96] and the mean difference between PP and RS conditions is 0.46 [89% CI: 0.11, 0.81).  Analysing the two factors (see Supplement S3) separately revealed that these results were driven primarily by the germ aversion factor as they did not replicate for the other (perceived infectability) factor. On the full dataset that combines Pilot data with the data from the actual experiment this pattern holds. The differences between conditions are, however, tiny, 0.15 [89% CI: -0.03, 0.33] between PP and Control and 0.33 [89% CI: 0.15, 0.51] between PP and RS.

Perceived RS, as indicated by RS scale scores, seemed to be relatively low in the RS condition. Contrary to expectations mean RS scale scores did *not* tend to be higher in the RS condition compared to the other conditions. In the pilot study, the mean difference between Control and RS conditions is 0.19 [89% CI: -0.12, 0.50] and the mean difference between PP and RS conditions is 0.18 [89% CI: -0.12, 0.48].  Analysing the three factors yielded by EFA (see Supplement S3) suggested that the only factor raised in the RS condition might be the perceived resource finiteness. When both datasets are analysed together, it becomes apparent that the Control condition peaks slightly above both experimental conditions (the difference in two factors – Worry about own future and Urge to act against eco-crisis – is more prominent). The difference between Control and PP is 0.12 [89% CI: -0.01, 0.24] and the difference between Control and RS is 0.13 [89% CI: 0.00, 0.26]. These unexpected results may be due to a compensatory reaction against the threatening video. For instance, it is possible that the participants who were exposed to the RS manipulation responded by thinking that their financial situation “could have been worse,” leading them to believe that their lives are relatively plentiful (in fact, it is commonly given advice in Turkish culture to engage in such downward comparison). Interestingly similar perspective seems to be offered by PP priming, but in both cases, the manipulation effect is extremely small (see Figure 2)^^[[1]](#footnote-1)^^.


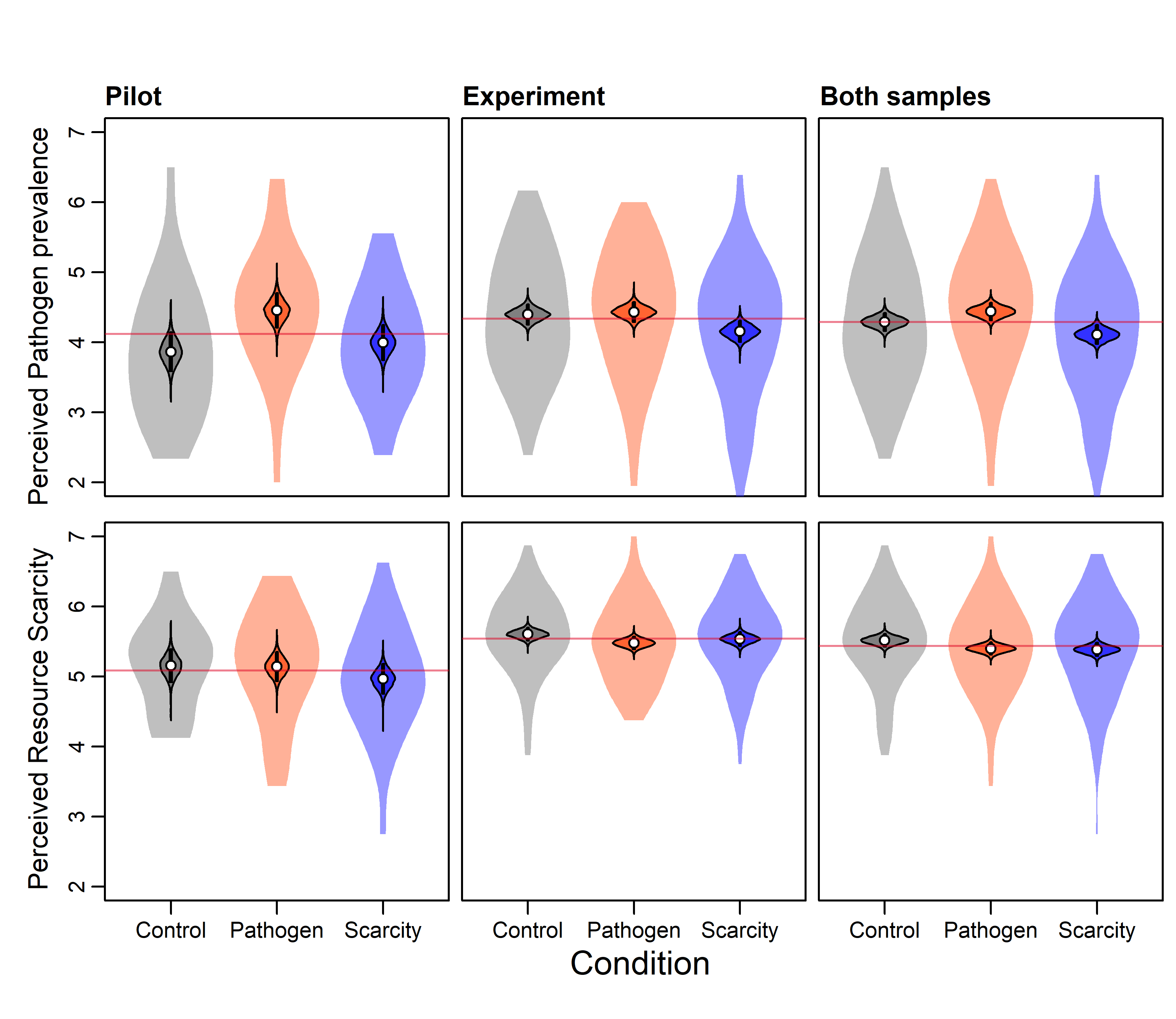


*Figure 2. Perceived PP and RS scores (calculated on a scale of standardized sum of 18 for PP and 16 for RS items, shown on a scale of average item) across conditions and samples. Semi-transparent violin plots outline the raw data distributions, contoured violin plots show the intercept posterior distributions, black rectangles span 89% of these distributions, and white points indicate their means. The pink line indicates the grand mean.*

The informal observations (notes written immediately after each participant completed the experiment and left the laboratory) of research assistants who conducted the pilot study indicate that participants in the two experimental conditions found the videos distressing.  Responses to the open-ended question about the effect of the video corroborate these observations. For instance, one participant in the RS condition reported that the video “reminded them of facts that they already knew but had forgotten due to the busy nature of daily life and made them worried for the future.”

Despite failing to obtain evidence that the RS scale scores were higher after watching the RS (versus another) video, other responses (e.g., on the video impact scale) independently suggested the effectiveness of the RS video.  Furthermore, it is not surprising that such abstract questions as employed in the RS scale are relatively insensitive to experimental primes – a situation we and others have observed in other contexts (Talhelm et al., 2015; Yilmaz & Saribay, 2017). Finally, the fact that we created this scale for the current purposes without extensive background psychometric work means that the scale itself may have failed to measure the intended construct properly.  That the PP scale scores showed evidence of video effectiveness (even if driven by one factor within that scale) also suggest a potential problem with the RS scale.

Overall, after these considerations, we decided that these results provided us with sufficient conviction that the videos we prepared were suitable for temporarily heightening concerns about PP and RS compared to the control prime.  In fact, considering the alternatives available in the literature, we think that the current method presents the best possibility for temporarily manipulating environmental harshness perceptions. Thus, we conducted the actual experiment using the same videos.

**S2: Environmental Harshness Perception Scale Items**

**Pathogen Prevalence Scale**

1. It really bothers me when people sneeze without covering their mouths.

2*. If an illness is ‘going around’, I will get it.

3. I am comfortable sharing a water bottle with a friend. (R)

4. I do not like to write with a pencil someone else has obviously chewed on.

5*. My past experiences make me believe I am not likely to get sick even when my friends are sick. (R)

6*. I have a history of susceptibility to infectious disease.

7. I prefer to wash my hands pretty soon after shaking someone’s hand.

8*. In general, I am very susceptible to colds, flu and other infectious diseases.

9. I dislike wearing used clothes because you do not know what the last person who wore it was like.

10+. Even if they have obviously been cleaned recently, I get uncomfortable using toilets that I know are used by other people, in places like hotels or homes.

11*. I am more likely than the people around me to catch an infectious disease.

12. My hands do not feel dirty after touching money. (R)

13*. I am unlikely to catch a cold, flu or other illness, even if it is ‘going around’. (R)

14+. Even if I know they have been cleaned, it makes me uneasy to use cutlery in places that serve the public, such as dining halls.

15. It does not make me anxious to be around sick people. (R)

16*. My immune system protects me from most illnesses that other people get. (R)

17. I avoid holding on to the handles in public transportation vehicles because of the risk that I may catch something from the previous user.

18+. It is not a problem for me to camp away from the hygienic facilities of daily life such as toilet paper, wet towels, and detergents (R)

*These items loaded onto the Perceived Infectability factor in Duncan et al (2009). The remaining items loaded onto the Germ Aversion factor in their study.

+ These items were newly written by the current authors.

Item 17 was slightly modified from its original form, which was as follows: “I avoid using public telephones because of the risk that I may catch something from the previous user.”

Items marked with (R) are reverse-coded.

**Resource Scarcity Scale**

1. If we use natural resources wisely, there is no reason why the world should not offer abundance to all mankind. (R)

2. I am not expecting to have a problem in meeting my basic needs such as food and water in my lifetime. (R)

3. As human civilization is becoming increasingly aware of environmental issues, we can witness that the “ecological crisis” we are supposedly experiencing is completely resolved in the period ahead of us. (R)

4. The number of people the planet can feed is limited and we are pushing this limit more and more.

5. Nature has always offered and will continue to offer abundance to humanity. (R)

6. We will suffer from the misuse of natural resources in the years to come.

7. The shortages of food and water that people in the lower levels of society are currently experiencing will soon begin to be experienced by the upper levels.

8. Food prices are constantly rising and I am concerned about that.

9. The decline in natural resources worries me about my near future.

10. I think I will personally be affected from the scarcity of basic resources such as water and food.

11. I think the presence of people in hunger is an element of natural balance. (R)

12. The uncontrolled growth of the population, especially in the last century, does not scare me. (R)

13. I believe that the accumulation of world’s population in certain countries and regions is a problem.

14. I care about doing something to prevent the careless depletion of natural resources.

15. I believe that society should be made more aware regarding the conservation of natural resources.

16. I think that even if our natural resources are almost exhausted, nature will soon be able to normalize this situation. (R)

Items marked with (R) are reverse-coded.

S3: Factors of PP and RS scale and experimental manipulation


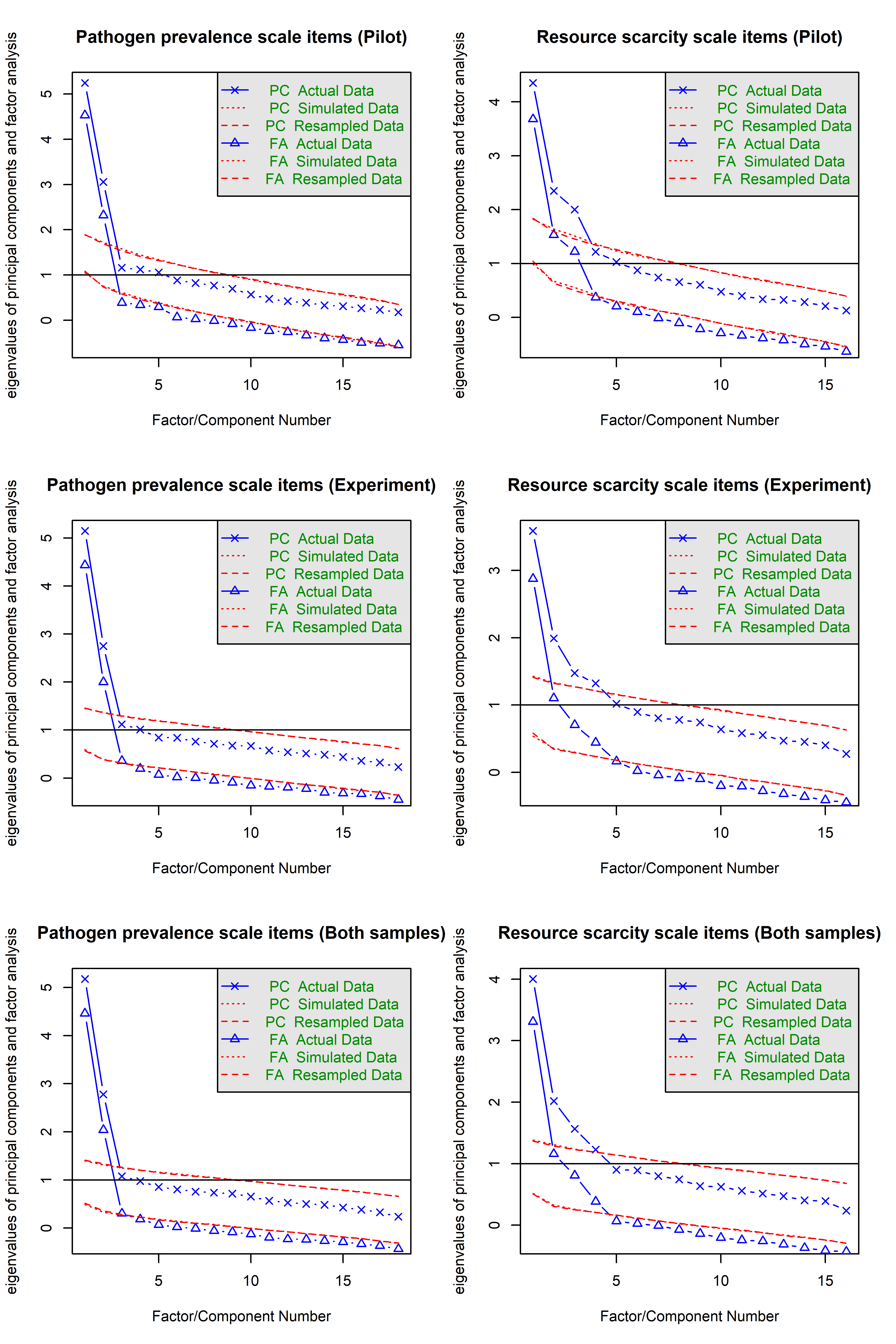


*Figure 3. Parallel analysis scree plots for the pilot sample, experimental sample, and both samples combined.*

Parallel analysis suggested that the number of factors underlying Pathogen Prevalence scale is 2 and number of factors underlying Resource Scarcity scale is 3 in the pilot study and 3 or 4 in the experimental data. Two factors for Pathogen prevalence and three factors of Resource scarcity scale were extracted in all samples using the exploratory factor analysis (EFA) with maximum likelihood extraction and promax rotation. The extracted factors were consistent in all samples. Factor loadings can be found in Table S1. Two factors of Pathogen prevalence; perceived infectability and germ aversion; replicated the two-factor structure reported in Duncan et al. (2009) exactly. Examining the content of items loading on three factors of Resource scarcity suggested that the first factor emphasizes worry about the future; the second one the felt need to act against an impending ecological crisis; and the third perception of the limited nature of planetary resources.

| **Table S1. Pathogen prevalence scale factor loadings** | | | | | | | |
| --- | --- | --- | --- | --- | --- | --- | --- |
|  |  | Pilot | | Experiment | | Both | |
| ID | Short item description | PI | GA | PI | GA | PI | GA |
| PP01 | Sneeze bothers me | -0.01 | **0.49** | 0.04 | **0.39** | 0.02 | **0.41** |
| PP02 | I will get ill | **0.4** | 0.2 | **0.48** | 0.19 | **0.47** | 0.2 |
| PP03 | Comfortable sharing bottle (R) | 0.02 | **0.57** | -0.19 | **0.51** | -0.15 | **0.52** |
| PP04 | Dislike chewed on pencil | 0.01 | **0.6** | 0.07 | **0.51** | 0.05 | **0.53** |
| PP05 | Not sick when friends sick (R) | **0.75** | -0.06 | **0.77** | -0.13 | **0.77** | -0.12 |
| PP06 | History of susceptibility | **0.64** | -0.01 | **0.72** | -0.13 | **0.7** | -0.1 |
| PP07 | Wash soon after shaking hands | -0.08 | **0.58** | -0.13 | **0.69** | -0.12 | **0.66** |
| PP08 | Susceptible to colds and flu | **0.96** | -0.21 | **0.92** | -0.11 | **0.93** | -0.14 |
| PP09 | Dislike wearing used clothes | -0.05 | **0.47** | 0.05 | **0.5** | 0.02 | **0.49** |
| PP10 | Uncomfortable using public toilets | -0.14 | **0.69** | 0.05 | **0.64** | 0 | **0.66** |
| PP11 | More likely to catch illness | **0.74** | 0.08 | **0.71** | 0.08 | **0.71** | 0.07 |
| PP12 | Hands do not feel dirty after money (R) | 0.17 | **0.43** | -0.08 | **0.52** | -0.03 | **0.5** |
| PP13 | Not likely to catch anything (R) | **0.8** | -0.09 | **0.66** | -0.02 | **0.69** | -0.03 |
| PP14 | Uneasy using restaurant cutlery | -0.02 | **0.64** | 0.01 | **0.58** | -0.01 | **0.6** |
| PP15 | Not anxious around sick (R) | 0.22 | **0.4** | 0.22 | **0.36** | 0.22 | **0.36** |
| PP16 | Immunity protects me (R) | **0.72** | -0.04 | **0.62** | 0.06 | **0.64** | 0.04 |
| PP17 | Avoid holding on handles | -0.11 | **0.75** | -0.03 | **0.67** | -0.05 | **0.69** |
| PP18 | Can camp away from toilet paper (R) | 0.05 | **0.42** | 0.11 | **0.35** | 0.1 | **0.37** |
|  |  |  |  |  |  |  |  |
| PI=Perceived infectability, GA=Germ aversion | | | | | | | |

| **Table S2. Resource scarcity scale factor loadings** | | | | | | | | | | |
| --- | --- | --- | --- | --- | --- | --- | --- | --- | --- | --- |
|  |  | Pilot | | | Experiment | | | Both | | |
| ID | Item | WF | AE | PF | WF | AE | PF | WF | AE | PF |
| RS01 | Wisdom offers abundance (R) | -0.19 | -0.03 | **0.47** | -0.19 | -0.23 | **0.45** | -0.18 | -0.18 | **0.46** |
| RS02 | Not expecting problems (R) | **0.72** | -0.29 | 0.1 | **0.61** | -0.2 | 0.25 | **0.65** | -0.21 | 0.15 |
| RS03 | Crisis will be resolved (R) | -0.1 | **0.38** | 0.28 | 0.1 | -0.06 | **0.44** | 0.04 | 0.04 | **0.43** |
| RS04 | We are pushing the limit | 0.27 | -0.05 | **0.66** | 0.01 | 0.07 | **0.46** | 0.12 | -0.05 | **0.54** |
| RS05 | Nature gonna deliver (R) | 0.21 | -0.12 | **0.79** | 0.12 | -0.07 | **0.57** | 0.13 | -0.16 | **0.59** |
| RS06 | We will suffer from misuse | 0.2 | **0.73** | -0.06 | 0.07 | **0.49** | 0.02 | 0.1 | **0.55** | 0.07 |
| RS07 | Shortage will affetc upper clesses | **0.44** | 0.18 | 0.03 | **0.47** | 0.13 | -0.02 | **0.48** | 0.13 | 0.01 |
| RS08 | Worried about rising prices | **0.42** | 0.14 | 0.1 | **0.32** | 0.23 | 0.08 | **0.4** | 0.16 | 0.13 |
| RS09 | Worried about nat. res. decline | **0.91** | 0.01 | -0.16 | **0.73** | 0.2 | -0.13 | **0.82** | 0.16 | -0.16 |
| RS10 | Personally affeced by scarcity soon | **0.91** | -0.07 | 0.07 | **0.94** | -0.21 | 0.03 | **0.9** | -0.11 | 0 |
| RS11 | People in hunger part of ballance (R) | -0.04 | **0.48** | -0.05 | -0.06 | **0.33** | 0.01 | -0.04 | **0.38** | 0.03 |
| RS12 | Uncontrolled growth does not scare (R) | 0.31 | 0.3 | **0.32** | 0.13 | 0.18 | **0.35** | 0.18 | 0.15 | **0.39** |
| RS13 | Accumulation of population is a problem | -0.11 | **0.42** | 0.15 | -0.08 | **0.2** | 0.12 | -0.09 | **0.25** | 0.2 |
| RS14 | Attempts to prevent depletion | 0.22 | **0.45** | -0.37 | 0.08 | **0.6** | -0.11 | 0.11 | **0.64** | -0.21 |
| RS15 | Society should wake up | -0.02 | **0.75** | -0.3 | -0.01 | **0.65** | 0 | -0.07 | **0.76** | -0.07 |
| RS16 | Nature will soon normalize situation (R) | -0.08 | **0.56** | 0.39 | -0.08 | 0.15 | **0.54** | -0.08 | 0.23 | **0.53** |
|  |  |  |  |  |  |  |  |  |  |  |
| WF=Worry about own future, AE=Urge to act against eco-crisis, PF=Perceived resource finiteness | | | | | | | | | | |

Analysis of the effect of experimental manipulation on the two factors of the PP scale revealed that changes in perceived PP are mostly due to changes in germ aversion, although in the experimental data, the perceived infectability was considerably lower in the RS condition than in the other two conditions as well (Figure 4).

Analysing the three factors of RS scale separately, we concluded that the observed elevation of RS scores was mostly due to the fear of own future and urge to act against imminent ecological crisis. Perhaps the video featuring planets and stars that was used as a control ignited the urge to protect the environment in our participants. The perceived finiteness of planetary resources was the only one that was overall higher in RS than the PP condition (Figure 5).


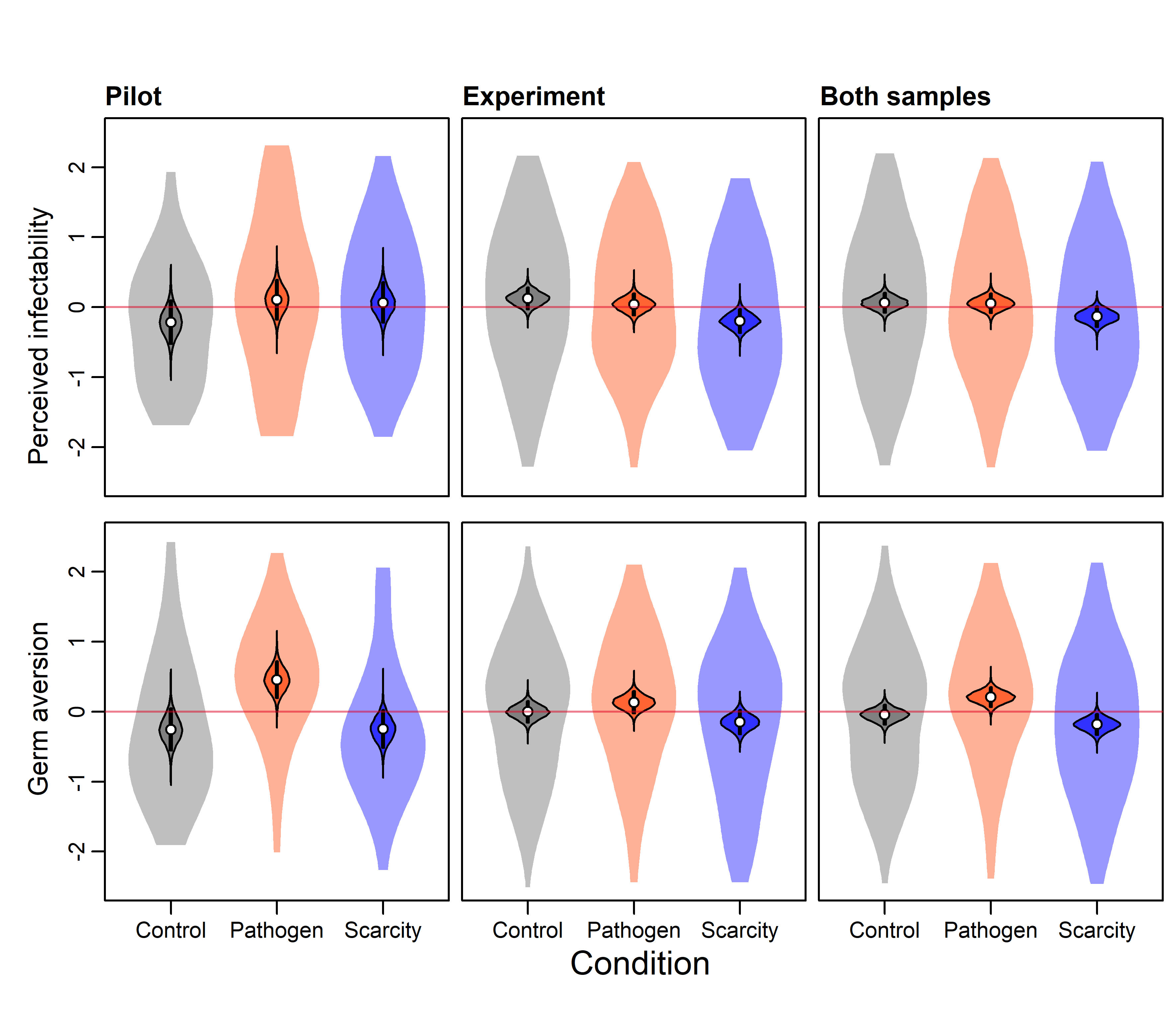


*Figure 4. Perceived infectability and Germ aversion calculated as standardized factor scores of Pathogen prevalence scale across conditions and samples. Semi-transparent violin plots outline the raw data distributions, contoured violin plots show the intercept posterior distributions, black rectangles span 89% of these distributions, and white points indicate their means. The pink line indicates the grand mean.*

*
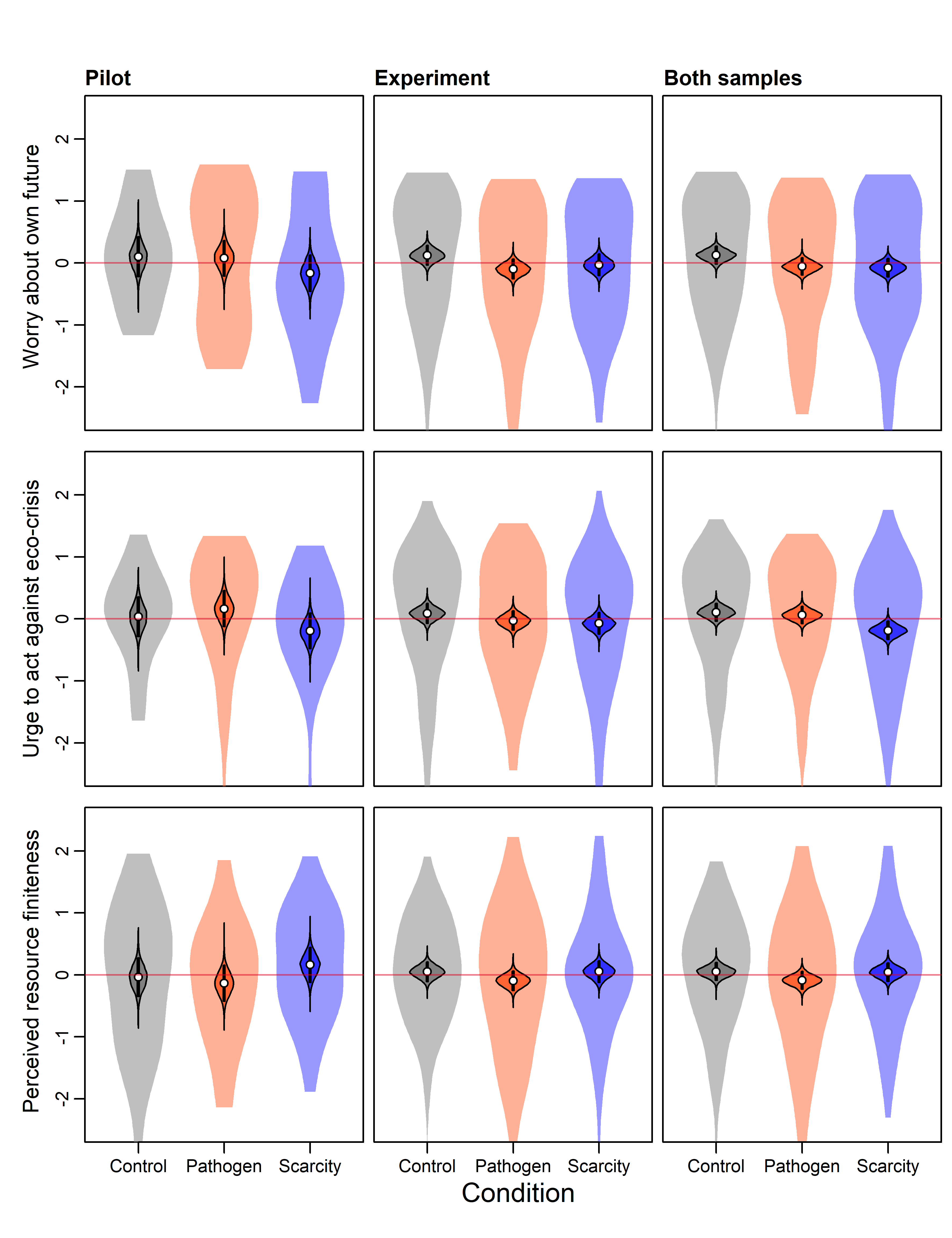
*

*Figure 5. Three factors of Resource scarcity scale labelled as Worry about own future, Urge to act against eco-crisis, and Perceived resource calculated as standardized factor scores across conditions and samples. Semi-transparent violin plots outline the raw data distributions, contoured violin plots show the intercept posterior distributions, black rectangles span 89% of these distributions, and white points indicate their means. The pink line indicates the grand mean.*

| S4: Overview of the Main Dependent Variables in the Experiment  **Table S3. Mean ratings (standard deviations) and selection probabilities in the Experiment** | | | | | | |
| --- | --- | --- | --- | --- | --- | --- |
| Condition | **Control** | | **Pathogen** | | **Scarcity** | |
| Photo | Feminized | Masculinized | Feminized | Masculinized | Feminized | Masculinized |
| Attractiveness ratings | 2.10 (1.41) | 2.17 (1.41) | 2.10 (1.39) | 2.13 (1.43) | 2.12 (1.44) | 2.17 (1.45) |
| Formidability ratings | 3.55 (1.42) | 4.01 (1.54) | 3.48 (1.47) | 3.97 (1.60) | 3.44 (1.47) | 3.90 (1.56) |
| Healthiness ratings | 3.88 (1.43) | 3.93 (1.39) | 3.88 (1.60) | 3.99 (1.59) | 3.71 (1.53) | 3.85 (1.54) |
| Selected as a more suitable long-term partner (%) | 48.53 | 51.47 | 52.04 | 47.96 | 48.74 | 51.26 |

S5: Structural model as a set of equations

**Photo selection**

$$\mathrm{SelectMasculinized}_{i}\sim\mathrm{Binomial}\left( 1,p_{i} \right)$$

$$p_{i}=\alpha_{COND[i]}+{\beta_{A}}_{COND[i]}{Adif}_{i}+{\beta_{F}}_{COND[i]}{Fdif}_{i}+{\beta_{H}}_{COND[i]}{Hdif}_{i}+{\beta_{PP}PPs}_{i}+{\beta_{RS}RSs}_{i}+{\beta_{R}side}_{i}+{z_{t}}_{TARGET[i]}\sigma_{t}+{z_{r}}_{RATER[i]}\sigma_{r}$$

**priors**

$$\alpha_{a}\sim\mathrm{Normal}(0,1.5)$$

$$\beta_{b}\sim\mathrm{Normal}(0,0.5)$$

**hyper-priors**

$$z_{j}\sim\mathrm{Normal}(0,1)$$

$$\sigma_{s}\sim\mathrm{Exponential}(0.5)$$

**Differences between ratings of masculinized and feminized photos**

**Attractiveness ratings**

$${Adif}_{i}={Amas}_{i}-{Afem}_{i}$$

$${Amas}_{i}\sim Ordered-logit({{\phi A}_{mas}}_{i},\kappa A)$$

$${Afem}_{i}\sim Ordered-logit({{\phi A}_{fem}}_{i},\kappa A)$$

$${{\phi A}_{mas}}_{i}={{\alpha A}_{mas}}_{COND[i]}+{{\beta A}_{masPP}PPs}_{i}+{{\beta A}_{masRS}RSs}_{i}+{{zA}_{t}}_{TARGET[i]}{\sigma A}_{t}+{{zA}_{r}}_{RATER[i]}{\sigma A}_{r}+{{zA}_{tr}}_{TARGET:RATER[i]}{\sigma A}_{tr}$$

$${{\phi A}_{fem}}_{i}={{\alpha A}_{fem}}_{COND[i]}+{{\beta A}_{femPP}PPs}_{i}+{{\beta A}_{femRS}RSs}_{i}+{{zA}_{t}}_{TARGET[i]}{\sigma A}_{t}+{{zA}_{r}}_{RATER[i]}{\sigma A}_{r}+{{zA}_{tr}}_{TARGET:RATER[i]}{\sigma A}_{tr}$$

**Formidability ratings**

$${Fdif}_{i}={Fmas}_{i}-{Ffem}_{i}$$

$${Fmas}_{i}\sim Ordered-logit({{\phi F}_{mas}}_{i},\kappa F)$$

$${Ffem}_{i}\sim Ordered-logit({{\phi F}_{fem}}_{i},\kappa F)$$

$${{\phi F}_{mas}}_{i}={{\alpha F}_{mas}}_{COND[i]}+{{\beta F}_{masPP}PPs}_{i}+{{\beta F}_{masRS}RSs}_{i}+{{zF}_{t}}_{TARGET[i]}{\sigma A}_{t}+{{zF}_{r}}_{RATER[i]}{\sigma F}_{r}+{{zF}_{tr}}_{TARGET:RATER[i]}{\sigma F}_{tr}$$

$${{\phi F}_{fem}}_{i}={{\alpha F}_{fem}}_{COND[i]}+{{\beta F}_{femPP}PPs}_{i}+{{\beta F}_{femRS}RSs}_{i}+{{zF}_{t}}_{TARGET[i]}{\sigma F}_{t}+{{zF}_{r}}_{RATER[i]}{\sigma F}_{r}+{{zF}_{tr}}_{TARGET:RATER[i]}{\sigma F}_{tr}$$

**Healthiness ratings**

$${Hdif}_{i}={Hmas}_{i}-{Hfem}_{i}$$

$${Hmas}_{i}\sim Ordered-logit({{\phi H}_{mas}}_{i},\kappa H)$$

$${Hfem}_{i}\sim Ordered-logit({{\phi H}_{fem}}_{i},\kappa H)$$

$${{\phi H}_{mas}}_{i}={{\alpha H}_{mas}}_{COND[i]}+{{\beta H}_{masPP}PPs}_{i}+{{\beta H}_{masRS}RSs}_{i}+{{zH}_{t}}_{TARGET[i]}{\sigma A}_{t}+{{zH}_{r}}_{RATER[i]}{\sigma H}_{r}+{{zH}_{tr}}_{TARGET:RATER[i]}{\sigma H}_{tr}$$

$${{\phi H}_{fem}}_{i}={{\alpha H}_{fem}}_{COND[i]}+{{\beta H}_{femPP}PPs}_{i}+{{\beta H}_{femRS}RSs}_{i}+{{zH}_{t}}_{TARGET[i]}{\sigma H}_{t}+{{zH}_{r}}_{RATER[i]}{\sigma H}_{r}+{{zH}_{tr}}_{TARGET:RATER[i]}{\sigma H}_{tr}$$

**priors**

$${\alpha X}_{a}\sim\mathrm{Normal}(0, 0.5)$$

$${\beta X}_{b}\sim\mathrm{Normal}(0, 0.5)$$

$$\kappa X\sim\mathrm{Normal}(0, 1.5)$$

**hyper-priors**

$${zX}_{j}\sim\mathrm{Normal}(0, 1)$$

$${\sigma X}_{s}\sim\mathrm{Exponential}(0.5)$$

**Standardized scores on Pathogen Prevalence and Resource Scarcity scales**

$${PPs}_{i}\sim\mathrm{Normal}({\alpha_{PP}}_{COND[i]}, \sigma_{PP})$$

$${RSs}_{i}\sim\mathrm{Normal}({\alpha_{RS}}_{COND[i]}, \sigma_{RS})$$

**priors**

$${\alpha_{PP}}_{COND[i]}\sim\mathrm{Normal}(0,1)$$

$${\alpha_{RS}}_{COND[i]}\sim\mathrm{Normal}(0,1)$$

$$\sigma_{PP}\sim\mathrm{Exponential}(1)$$

$$\sigma_{RS}\sim\mathrm{Exponential}(1)$$

S6: Comparison of prior and posterior parameter distributions


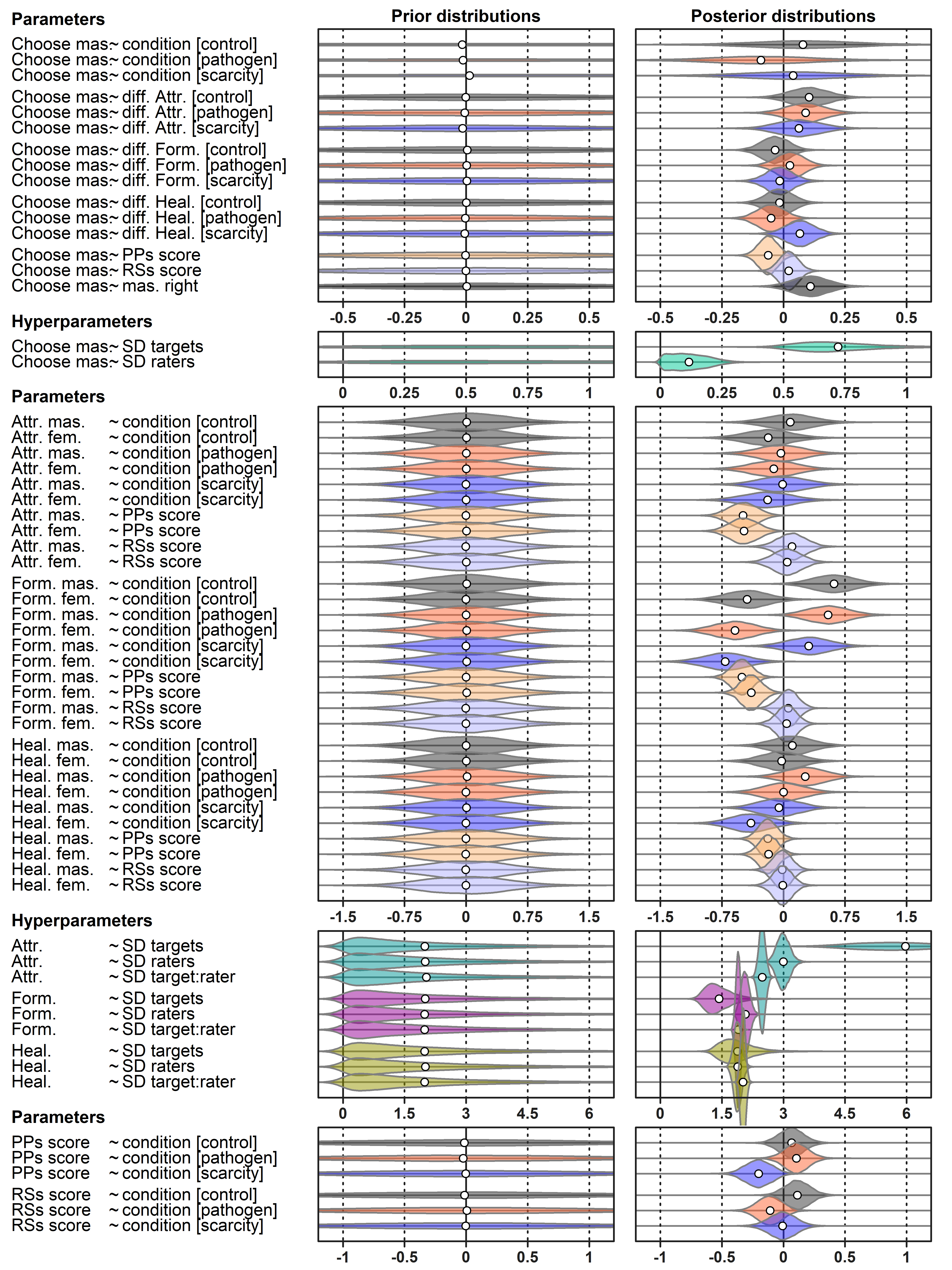


*Figure 6. Prior and posterior distribution of parameter values, white points indicate distribution means. Both distribution sets are based on 10 000 samples from given joint distributions.*

S7: Posterior distributions for alternative inclusion criteria


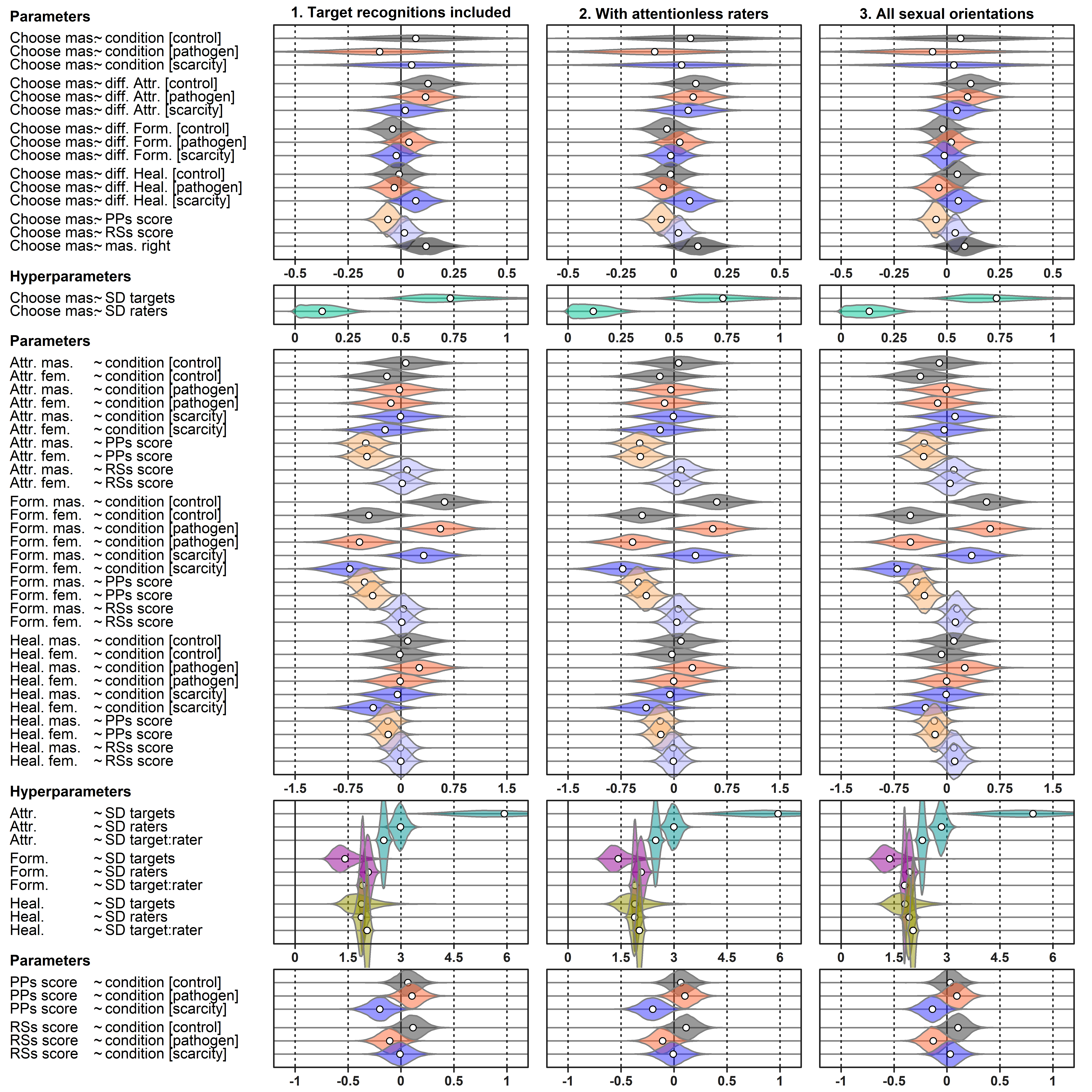


*Figure 7. Alternative posterior distributions. All inclusion criteria converge on similar joint posterior distribution. Choices regarding the sample therefore have no influence over the findings reported in the article. All visualizations are based on 10 000 samples from joint posterior distributions.*

S8: Predicted Mate Preferences

1. Predicted mate preferences assuming all masculinized photographs on right side


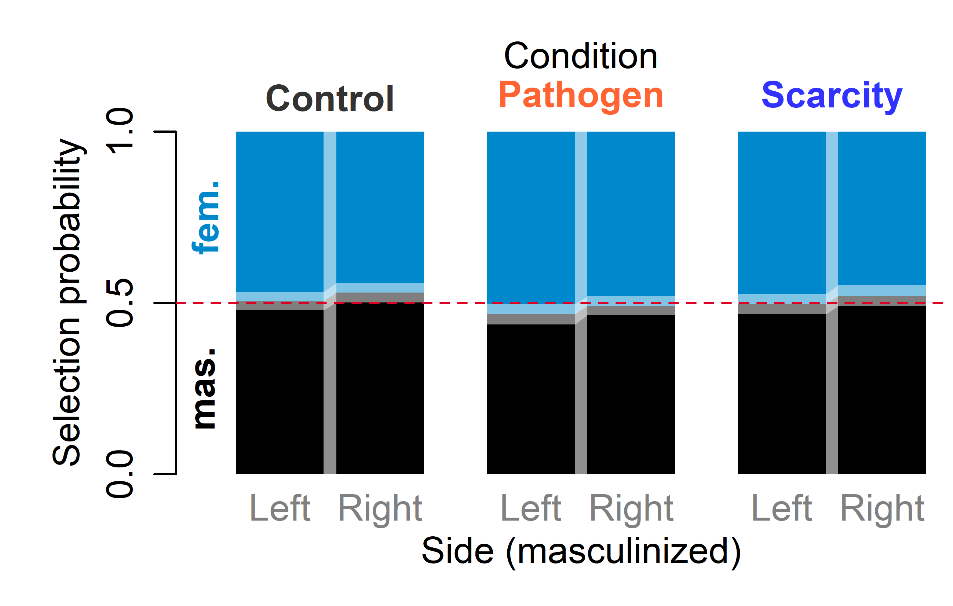


*Figure 8. The predicted proportion of long-term partner selection if all masculinized photographs are presented on one side (left or right) and all participants are shown the same priming video (Control, Pathogen, or Scarcity). Perceived PP, RS, and all rating differences are held constant at 0. Varying intercepts from the sampled posterior distributions are used. Color boundary indicates the median probability, light rectangles envelope 89% compatibility intervals of predicted probabilities.*

b. Predicted mate preferences in the control condition by perceived environmental harshness


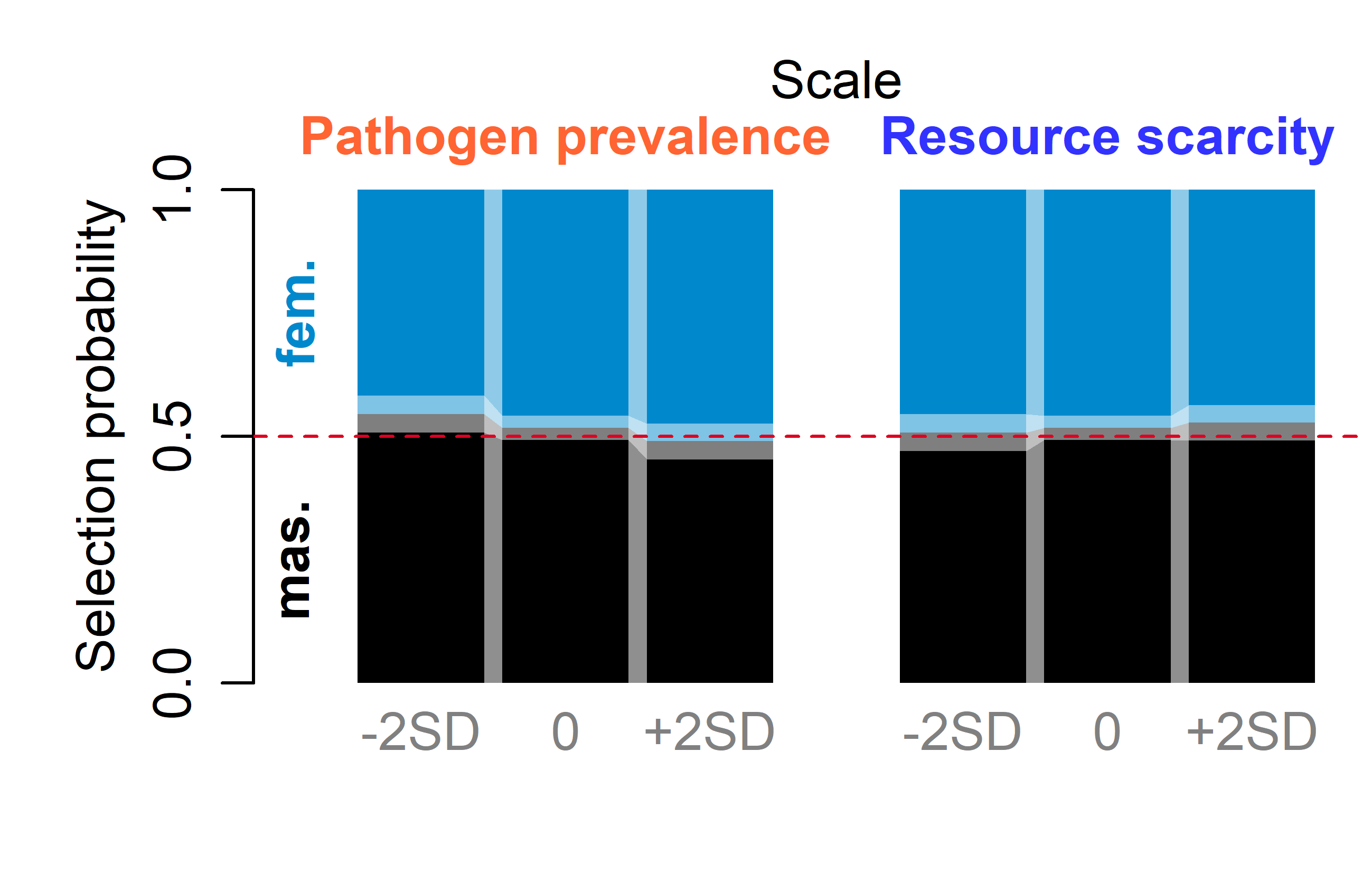


*Figure 9. The predicted proportion of long-term partner selection in the control condition by perceived PP and RS scores. The display laterality is neglected (the contrast assumes a 0 value). All rating differences and the complementary scale are held constant at 0. Varying intercepts from the sampled posterior distributions are used. Boundary indicates the median probability, light rectangles envelope 89% compatibility intervals of predicted probabilities.*

c. Predicted mate preferences by experimental condition and rating difference


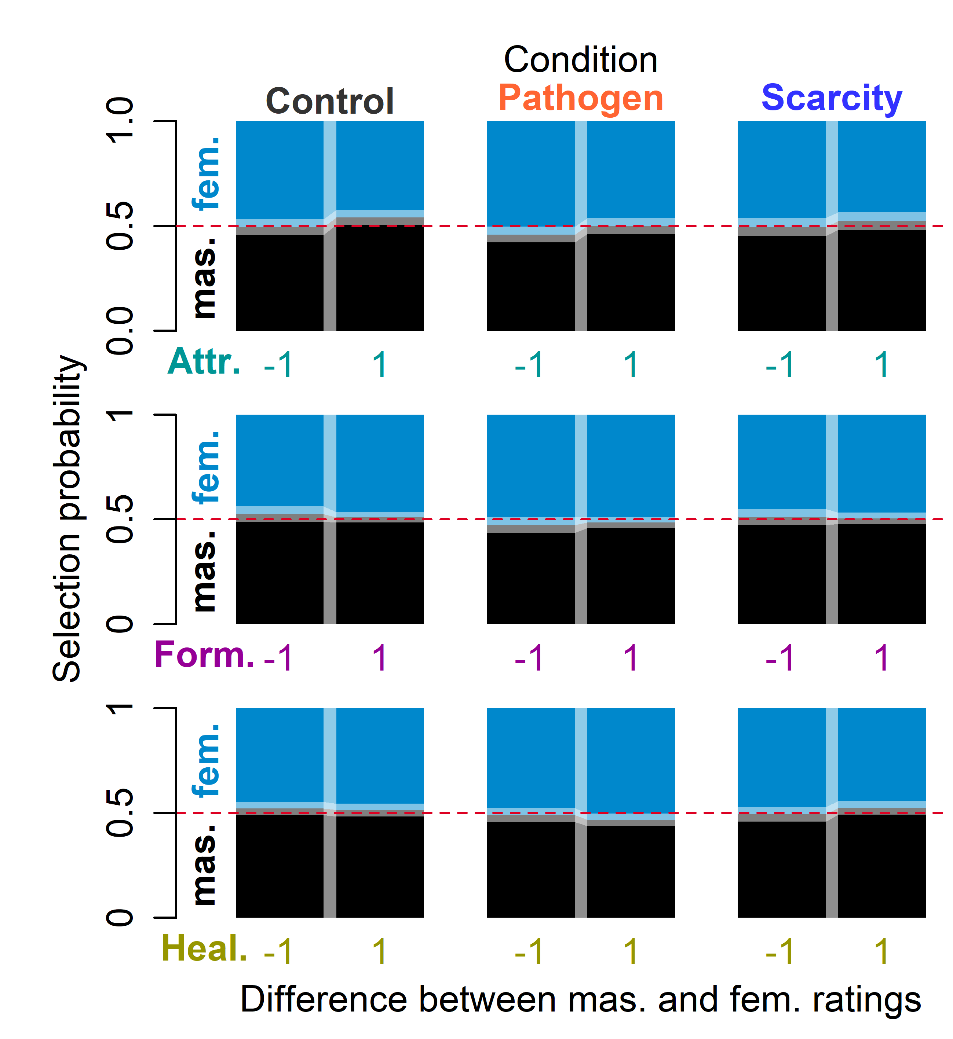


*Figure 10. The predicted proportion of long-term partner selection by condition and rating difference. All other rating differences than the one in question, perceived PP and RS scores, and laterality contrast are held constant at 0. Varying intercepts from the sampled posterior distributions are used. Boundary indicates the median probability, light rectangles envelope 89% compatibility intervals of predicted probabilities.*

S9: Predicted Ratings


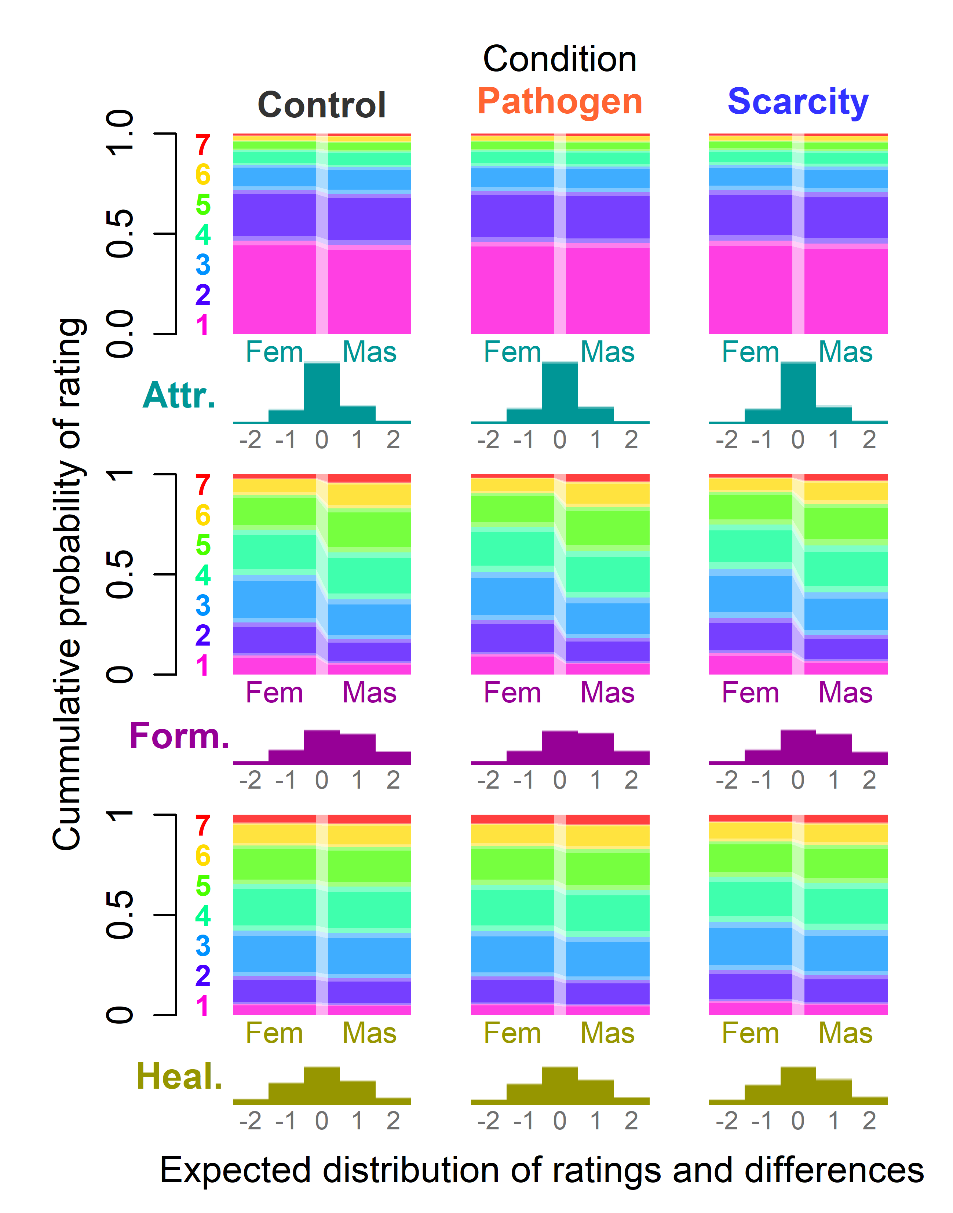


*Figure 11. The predicted ratings and differences between them. Perceived PP and RS scores are held constant at 0. Varying intercepts from the sampled posterior distributions are used. Boundaries indicate median cumulative probabilities, light rectangles envelope 89% compatibility intervals of predicted probabilities. Differences are represented by histograms below visualization of paired ratings. Differences > 2 in absolute values are omitted for clarity.*

S10: Variation in model’s predictive accuracy

The model including varying intercepts predicted the choice correctly in 62.6 % of cases [89% CI: 61.8, 63.4]. The variation in predictive precision between data points was, however, huge. The varying intercepts were vital for the reported model precision. There was a large amount (47.4 %) of target-rater combinations for which the choice was a sure bet (more than 90% of posterior samples predicted the choice correctly), while the second most frequent option was that the model had almost no chance of predicting the choice well (22.8 % of data with less than 10% probability of success), with very few data in between those extremes (see Figure 12). These “sure misses” represent counterexamples to the huge agreement between raters in terms of whether feminization or masculinization suits given target better, and no model with a multilevel structure, without a key to raters’ preference can predict them well.

The naive model that ignores all information about differences between targets and raters (a model that aims to predict a choice of an unknown rater choosing between photos on an unknown target) barely outperforms blind guessing with a mean success rate of 51.3 % [89% CI: 50.1, 52.5 %]. Sure hits (3.4%) and sure misses (2.4%) are, understandably, almost absent with this prediction engine (See Figure 12).


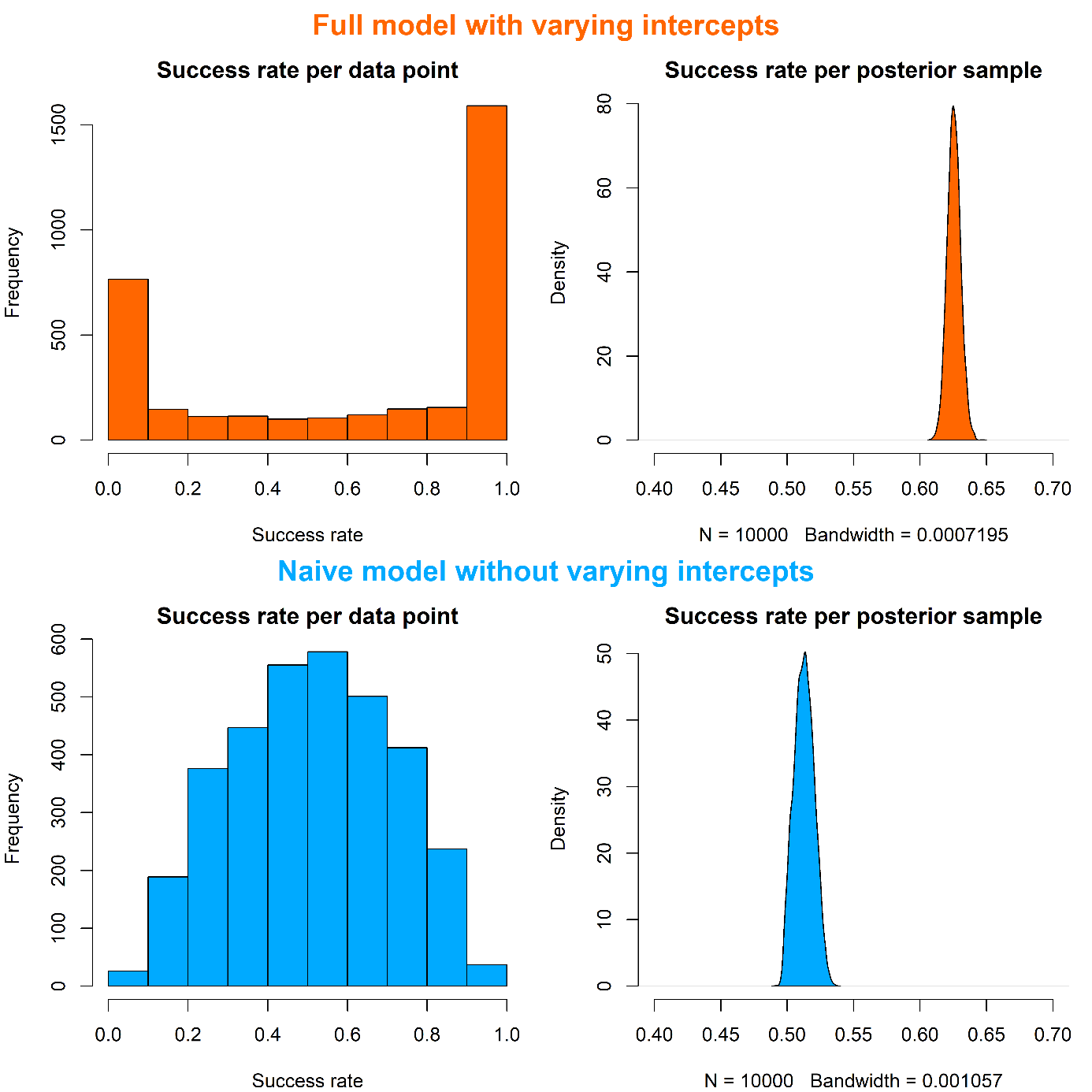


*Figure 12. Prediction success per datum and sample for two models – model with varying intercepts from sampled posterior (upper panel) and model trying to predict a choice in an unknown combination of target and rater from the predictor variables alone (lower panel).*

The probability of masculinized face being chosen was 0.52 [89% CI: 0.45, 0.58] in the Control condition, 0.48 [89% CI: 0.41, 0.55] in the Pathogen Prevalence condition, and 0.51 [89% CI: 0.45, 0.58] in the Resource scarcity condition. The posterior distribution of log-odds difference between Control and Pathogen Prevalence condition was 0.16 [89% CI 0.02, 0.30], 0.01 [89% CI -0.14, 0.16] between Control and Resource Scarcity, and 0.15 [89% CI -0.00, 0.29] between Resource Scarcity and Pathogen Prevalence. The posterior distribution of total effects is in Figure 15, the predictions for each combination of effect and presentation laterality in Figure 16.


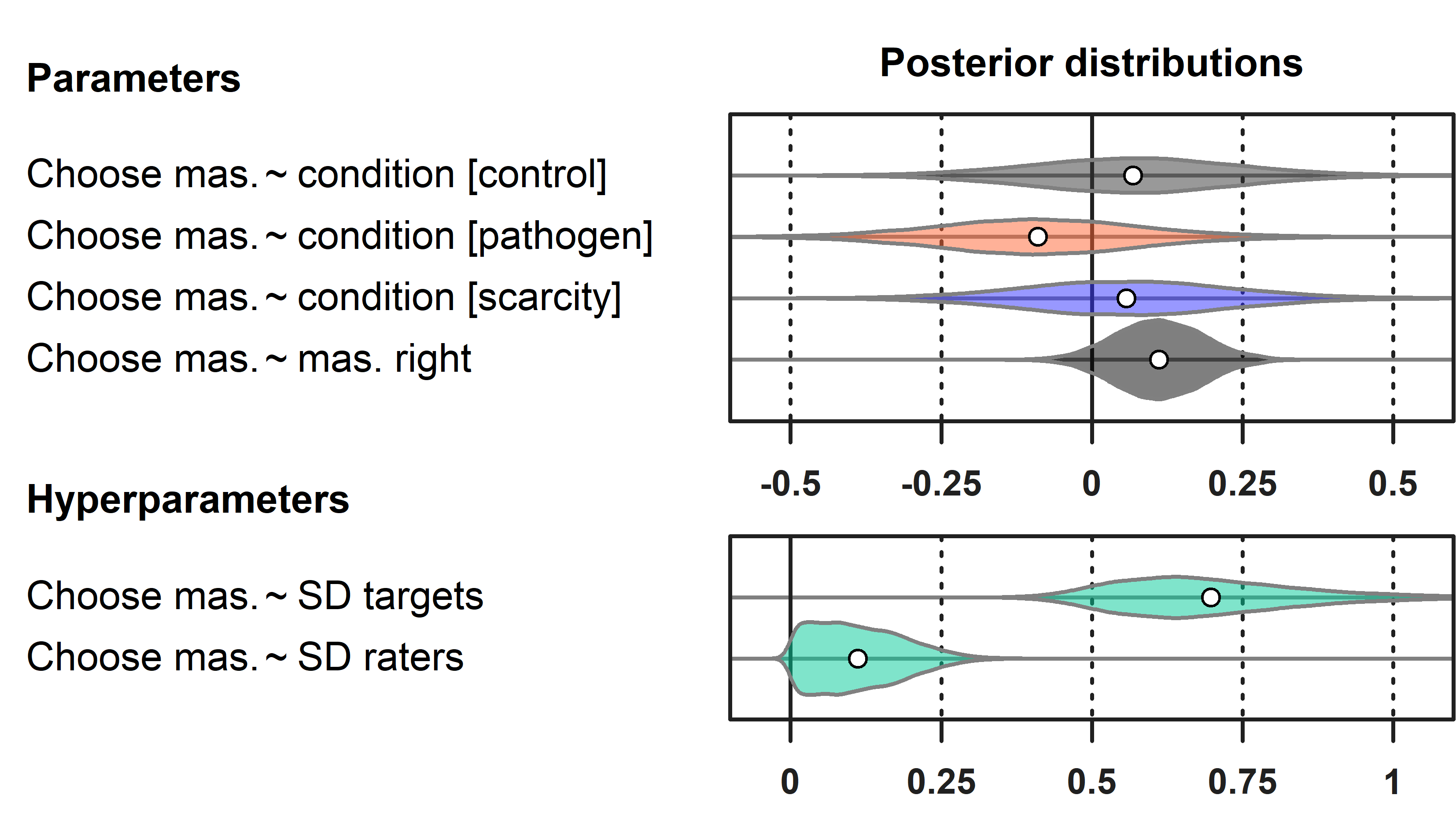


*Figure 13. The posterior distribution of total condition effects and varying intercepts of raters and targets in the simplified model.*


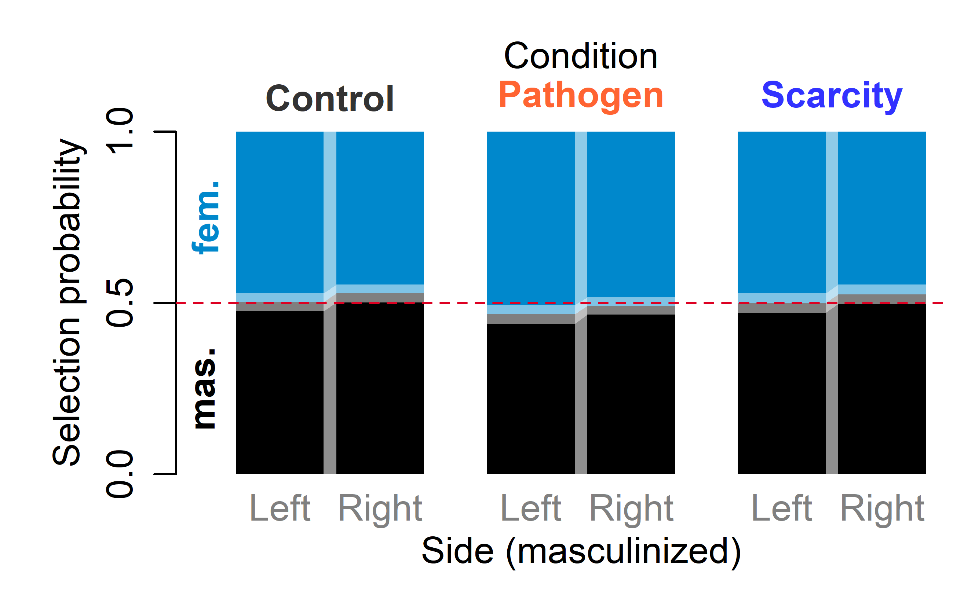


*Figure 14. The predicted proportion of long-term partner selection if all masculinized photographs are presented on one side (left or right) and all participants are shown the same priming video (Control, Pathogen, or Scarcity). Total effects are displayed. Colour boundary indicates the median probability, light rectangles envelope 89% compatibility intervals of predicted probabilities.*

S11: Association of Pathogen Prevalence and Resource Scarcity Scale Scores with Facial Ratings

Participants with high self-report PP scores were reluctant to use high attractiveness ratings. If all participants (we report predictions for Control condition only) scored 2 SD below the average PP score, the mean rating would be 2.41 [89% CI: 2.25, 2.58] for masculinized and 2.34 [89% CI: 2.18, 2.50] for feminized photograph, while if all scored 2 SD above the average PP score, we would expect mean ratings 1.95 [89% CI: 1.83, 2.08] for masculinized and 1.90 [89% CI: 1.78, 2.03] for feminized photos. The expected difference between ratings in a target:rater set would be 0.07 [89% CI: 0.01, 0.14] in the former and 0.05 [89% CI: 0.00, 0.10] in the later.

RS scores had little association with mean attractiveness ratings. If all participants (predicted for Control condition) scored 2 SD below the average RS score, the mean rating would be 2.12 [89% CI: 1.98, 2.27] for masculinized and 2.09 [89% CI: 1.95, 2.23] for feminized photograph, while if all scored 2 SD above the average RS score, mean ratings would increase only slightly to 2.22 [89% CI: 2.08, 2.36] for masculinized and 2.13 [89% CI: 1.99, 2.27] for feminized photos. The expected difference between ratings in the former would be 0.03 [89% CI: -0.02, 0.09] and 0.09 [89% CI: 0.03, 0.15] in the latter, which suggests that the rated attractiveness of masculinized face might grow disproportionally to the attractiveness of the feminized photo with increasing perceived RS.

Ratings of formidability changed with perceived PP and RS in a similar fashion as ratings of attractiveness. RS scores had little impact on formidability ratings and differences between them. Healthiness ratings declined as perceived PP increased, but the difference between perceived healthiness of masculinized and feminized photos remained constant. RS scores did not influence healthiness ratings and differences between them. All rating predictions are summarized in Figure 15.

To investigate a potential causal link between formidability, healthiness, and attractiveness, we evaluated a model of an alternative causal core with attractiveness as a function of two ordered categorical predictors: formidability and healthiness rating. The model (see Supplement S11) included also the contrast between masculinized and feminized version of the same photograph and varying intercepts of raters, targets, and interaction between them.

In all three conditions, perceived healthiness was a stronger predictor of rated attractiveness than rated formidability. Both were, however, associated with attractiveness positively. The association between formidability and attractiveness was similarly strong in all three conditions, perhaps with a slight decrease in PP condition. Contrary to our expectations, the relationship between healthiness and attractiveness was similarly pronounced across conditions [0.30, 89% CI: -0.41, 1.02 posterior difference between slopes in Control and PP, and 0.28, 89% CI: -0.45, 1.03 between RS and PP]. Perhaps the masculinity of faces that elevates both rated formidability and healthiness discouraged participants in PP condition from giving high ratings. The posterior distributions are still very broad, so it might seem too hasty to draw any strong conclusions from them. It is, however, very unlikely (see posterior distributions in Figure 16) that PP condition elicits unusually strong relationship between rated healthiness and attractiveness, which was hypothesized earlier. The predictions of mean rated attractiveness given formidability and healthiness are summarized in Figure 17.


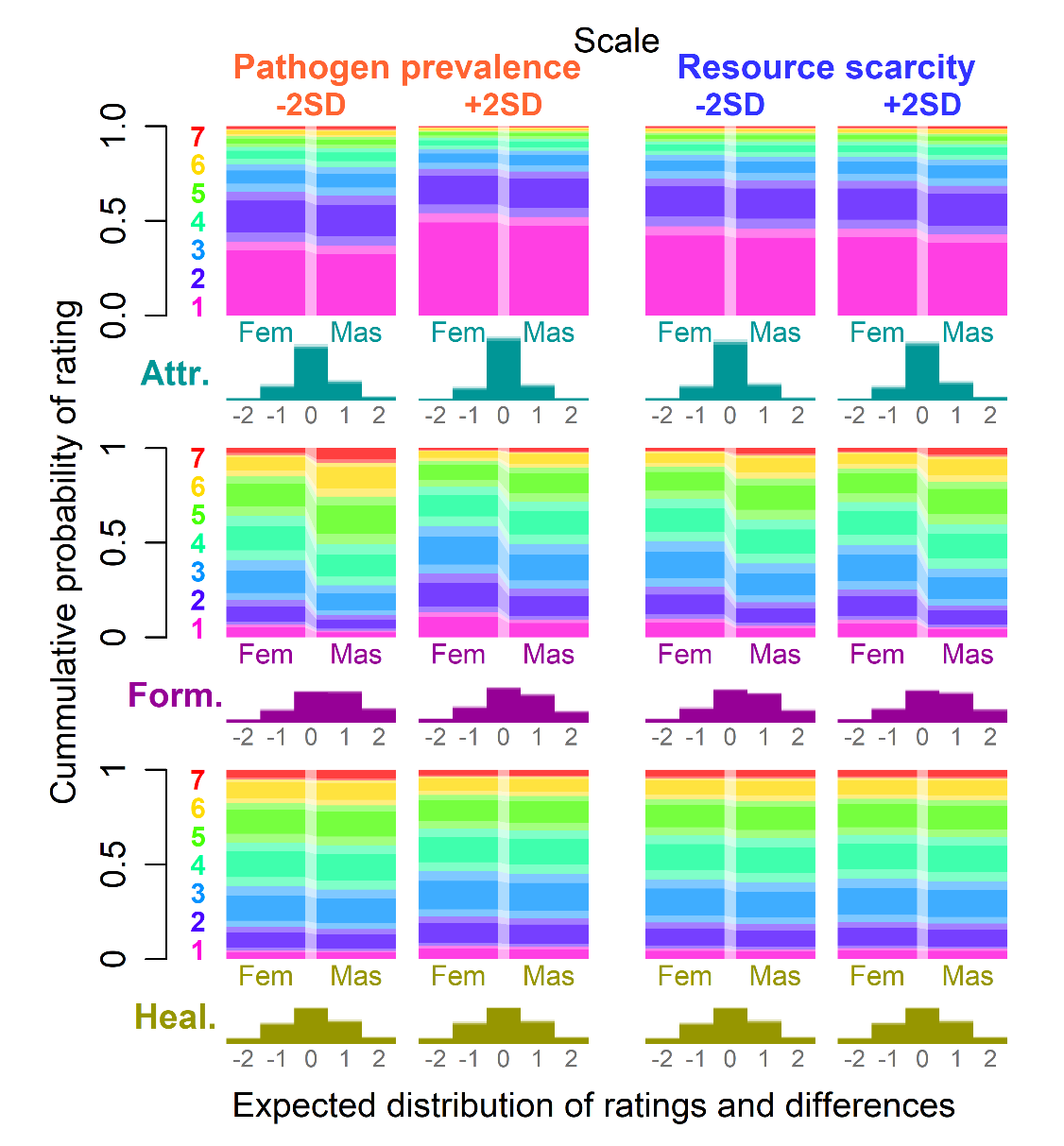


*Figure 15. Predicted ratings and differences between them based on extreme PP and RS scores in the Control condition. Varying intercepts from the sampled posterior distributions are used. Boundaries indicate median cumulative probabilities, light rectangles envelope 89% compatibility intervals of predicted probabilities. Differences are represented by histograms below visualization of paired ratings. Differences >2 in absolute values are omitted for clarity.*

PP priming did not elevate perceived PP scores by much. The expected difference between intercepts on standardized PP scale in Control and PP conditions was -0.04 [89% CI: -0.26, 0.18] (-0.04 [89% CI: -0.23, 0.16] on the scale of item average), the perceived PP, however, dropped in the RS condition, the difference between Control and RS was 0.27 [89% CI: 0.03, 0.50] (0.24 [89% CI: -0.03, 0.45] on the scale of item average). The perceived RS was highest in the Control condition. The expected difference between Control and PP conditions was 0.22 [89% CI: -0.01, 0.45] (0.13 [89% CI: -0.00, 0.26] on the scale of item average) and between Control and RS conditions it was 0.12 [89% CI: -0.11, 0.36] (0.07 [89% CI: -0.06, 0.20] on the scale of item average). There was still a lot of variation in PP and RS scales that could not be explained by the priming condition. The standard deviation of the distribution around the by-condition intercept was 1.00 [89% CI: 0.93, 1.07] on PP scale and 1.00 [89% CI: 0.94, 1.07] on RS scale, almost like there was no difference between conditions.

S12: Model of attractiveness as a function of formidability and healthiness

$${Amas}_{i}\sim Ordered-logit({{\phi A}_{mas}}_{i},\kappa A)$$

$${Afem}_{i}\sim Ordered-logit({{\phi A}_{fem}}_{i},\kappa A)$$

$${{\phi A}_{mas}}_{i}={{0.5\beta}_{M}}_{COND[i]}+{\beta_{F}}_{COND[i]}\sum_{j=0}^{{Fmas}_{i}-1} {\delta F}_{j}+{\beta_{H}}_{COND[i]}\sum_{j=0}^{{Hmas}_{i}-1} {\delta H}_{j}+{{zA}_{t}}_{TARGET[i]}{\sigma A}_{t}+{{zA}_{r}}_{RATER[i]}{\sigma A}_{r}+{{zA}_{tr}}_{TARGET:RATER[i]}{\sigma A}_{tr}$$

$${{\phi A}_{fem}}_{i}={{-0.5\beta}_{M}}_{COND[i]}+{\beta_{F}}_{COND[i]}\sum_{j=0}^{{Ffem}_{i}-1} {\delta F}_{j}+{\beta_{H}}_{COND[i]}\sum_{j=0}^{{Hfem}_{i}-1} {\delta H}_{j}+{{zA}_{t}}_{TARGET[i]}{\sigma A}_{t}+{{zA}_{r}}_{RATER[i]}{\sigma A}_{r}+{{zA}_{tr}}_{TARGET:RATER[i]}{\sigma A}_{tr}$$

**priors**

$$\beta_{b}\sim\mathrm{Normal}(0, 1)$$

$$\delta X\sim\mathrm{Dirichlet}(\{2,2,2,2,2,2\})$$

$$\kappa X\sim\mathrm{Normal}(0, 1.5)$$

**hyper-priors**

$${zA}_{j}\sim\mathrm{Normal}(0, 1)$$

$${\sigma A}_{s}\sim\mathrm{Exponential}(0.5)$$

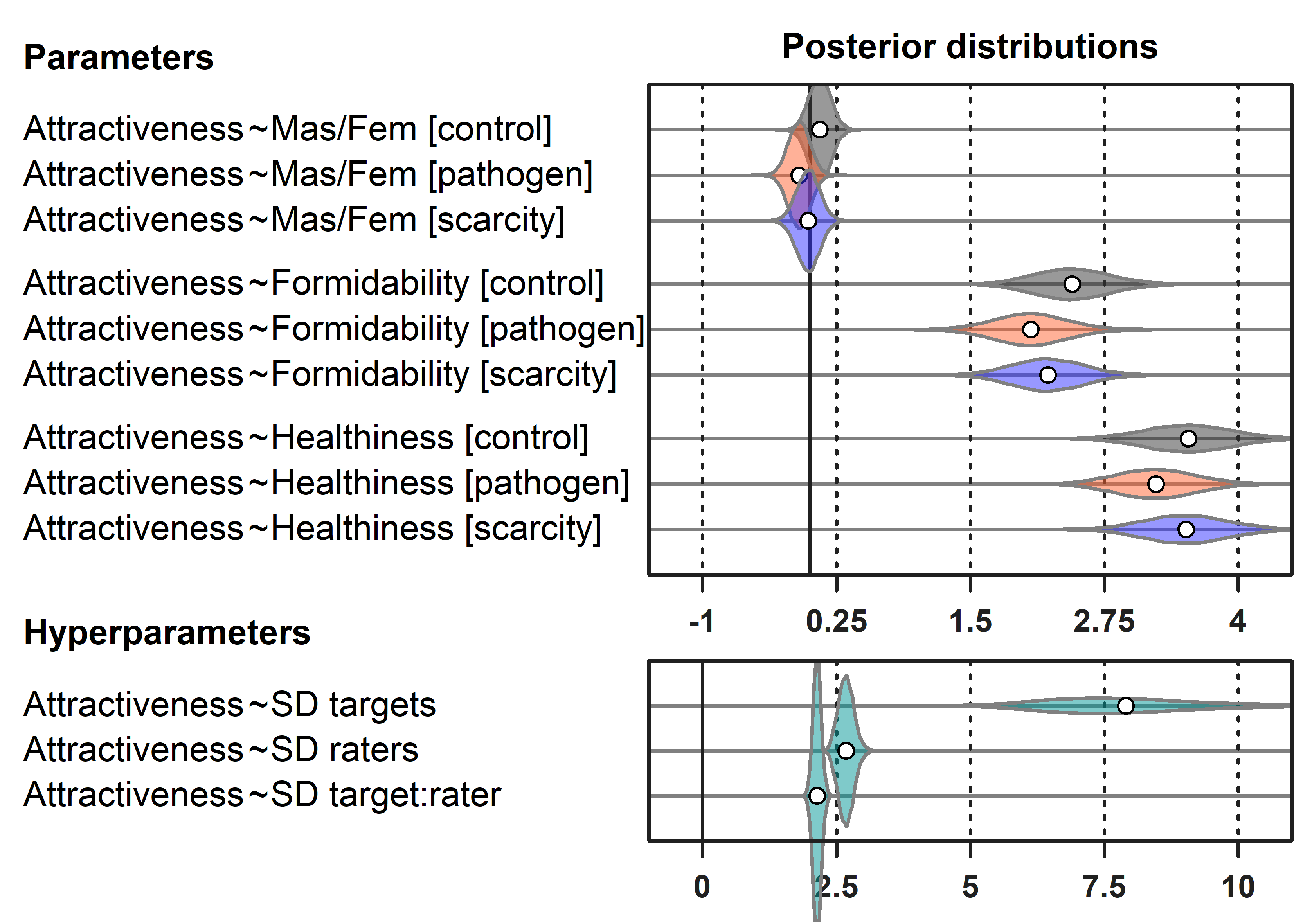


*Figure 16. Posterior distribution of parameter values in a model of attractiveness as a function of formidability and healthiness where independent slopes are estimated within each condition. Parameter values of ordered categorical predictors indicate the log-odds difference between the lowest (1) and highest (7) predictor (formidability or healthiness) ratings.*


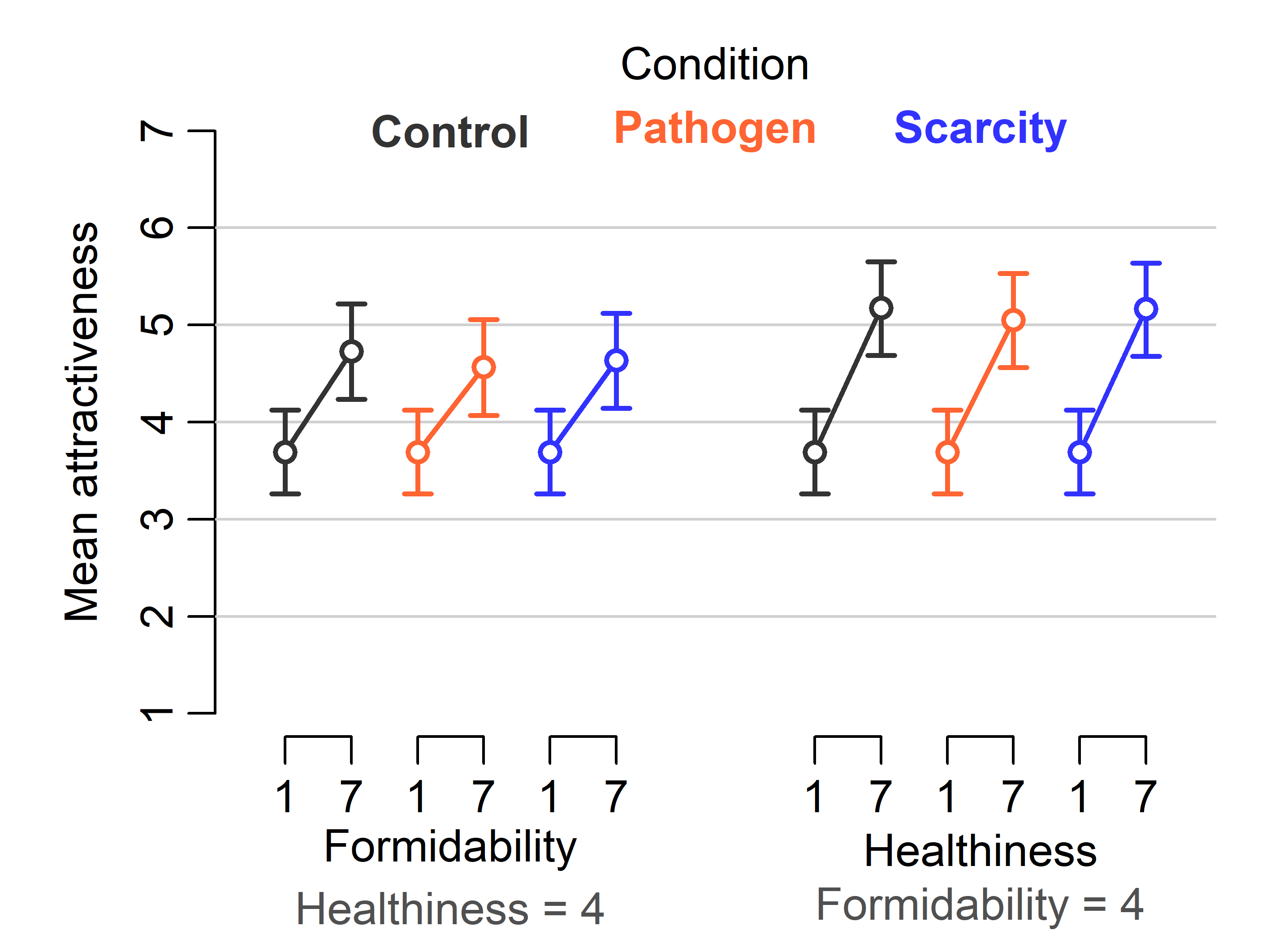


*Figure 17. Predictions of mean attractiveness rating based on samples from joint posterior distribution of parameter values of causal model of attractiveness as a function of formidability and healthiness. Feminization/masculinization is ignored (the contrast assumes a 0 value), varying intercepts are ignored. The complementary predictor is held constant at 4, which is a median formidability and healthiness value. The error bars envelope 89% CI of a mean expected rating.*

1. We also observed a difference of 0.45 [89% CI: 0.31, 0.59] in grand mean of RS scores between the RS condition of the pilot study and the actual experiment. This may indicate a shift in the economic situation of an average Turkish student across the periods in which these two data set were collected, but this is not highly likely because data collection was separated by only a few months. [↑](#footnote-ref-1)
